# Supplementary material for: Condensate droplet roaming on nanostructured superhydrophobic surfaces
Source: Nat Commun. 2025 Jan 30;16:1167. doi: 10.1038/s41467-025-56562-x (PMC11782698; doi:10.1038/s41467-025-56562-x)
Supplement: Supplementary file 1 — Supplementary Information [file 41467_2025_56562_MOESM1_ESM.pdf]

Supplementary information for

Condensate droplet roaming on nanostructured superhydrophobic surfaces

Cheuk Wing Edmond Lam[1, †], Kartik Regulagadda[1, ‡], Matteo Donati[1], Abinash Tripathy[1], Gopal Chandra Pal[2], Chander Shekhar Sharma[2], Athanasios Milionis[1], and Dimos Poulikakos[1, *]

[1] Laboratory of Thermodynamics in Emerging Technologies, Department of Mechanical and Process Engineering, ETH Zurich, Sonneggstrasse 3, 8092 Zurich, Switzerland

[2] Thermofluidics Research Lab, Department of Mechanical Engineering, Indian Institute of Technology Ropar, Rupnagar, Punjab, 140001 India

[†] Present address: Department of Mechanical Engineering, Massachusetts Institute of Technology, 77 Massachusetts Avenue, Cambridge, MA 02139, United States

[‡] Present address: 3114, Etcheverry Hall, MTSN, Department of Mechanical Engineering, UC Berkeley, CA 94720, United States

[*] Corresponding author

Prof. Dr. Dimos Poulikakos

Email: dpoulikakos@ethz.ch

Phone: +41 44 632 27 38

Fax: +41 44 632 11 76

Table of contents

S1. Thickness of the pPFDA layer by ellipsometry

S2. Condensation and observation setup

S3. Effect of microstructures on droplet motion

S4. Image processing, droplet measurement and event tracking

S5. Roaming on various nanostructured surfaces

S6. Evolution of roaming events

S7. Real roaming velocity

S8. Additional heat transfer measurements

S9. Rates of condensate volume removal and surface area renewal

S10. Critical nucleation diameter and transition subcooling

S11. Volumetric nucleation rate and cavity filling timescale

S12. Effects from droplet size mismatch

S13. Stages of x-momentum generation

S14. Dewetting and the efficiency in kinetic energy conversion

S15. Setup of numerical simulation cases

S16. Roaming on hierarchical surfaces

S1. Thickness of the pPFDA layer by ellipsometry

The thickness of the pPFDA layer is measured by ellipsometry (V-VASE, J.A. Woollam), using a reference silicon wafer coated with the same iCVD process as the samples. Specifying the material stack, data fitting is performed with the software provided by the company to determine the layer thickness. Measurement is taken for at least two locations on each sample for the mean, over wavelengths of 200 – 1600 nm for at least three different angles.

The native oxide thickness of silicon is first determined to be 1.7 nm with a material stack of silicon (525 μm) and silicon dioxide, using a sample cut from a pristine silicon wafer. Next, the pPFDA layer is added to the material stack as a Cauchy material, using constants A: 1.3992, B: 0.0069215, C: -0.00024462, which are found to provide the best fit. The thickness of the pPFDA layer is determined to be 3.5 nm. An example for the data fitting at one location can be found in **Supplementary Figure 1**.

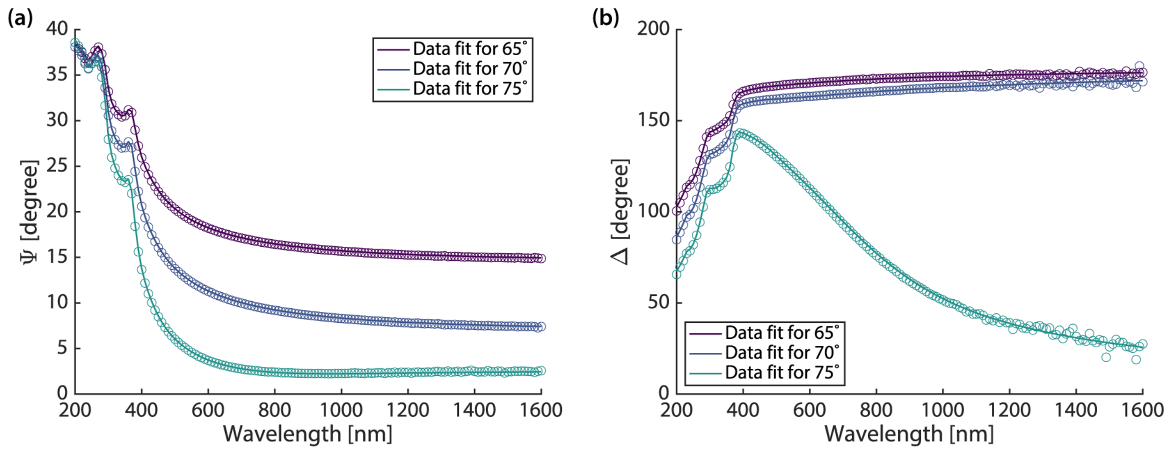

Supplementary Figure 1: Raw ellipsometer measurements (circles) and their fit to the model (solid lines) at one location of a pPFDA-coated silicon wafer. Each colour represents measurement at one angle. (a) Spectra of the amplitude component Ψ and (b) the phase difference Δ. Source data are provided as a Source Data file.

S2. Condensation and observation setup

Condensation setup

The condensation setup is similar to our previous work.[1,2] Condensation is performed with saturated steam at a nominal pressure of 30 mbar. The condensation chamber is installed as a part of an open system consisting of a pressure and steam source, the boiler, and a pressure sink, the vacuum pump. The schematic of the system is shown in **Supplementary Figure 2**.

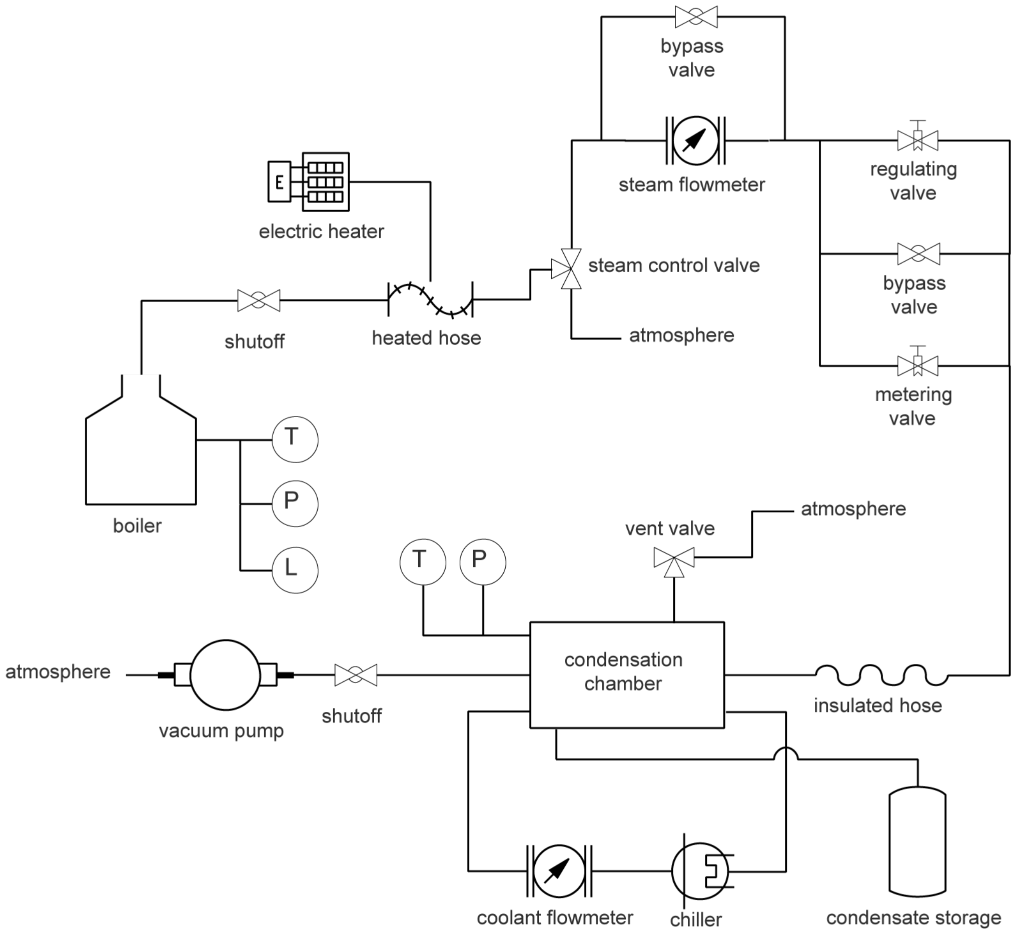

Supplementary Figure 2: System consisting of the condensation chamber. Reproduced from our previous work[2] which is under the Creative Commons licence CC BY-NC-ND 4.0.

During operation, a boiler containing deionised water is used to generate steam at 1.4 ± 0.01 bar. The steam passes through a heated hose, and its flow rate is measured by a flowmeter (FAM3255, ABB). Regulating (SS-6BMRG-MM, Swagelok) and metering (SS-6BMW-MM, Swagelok) valves are used to control the steam flow so that the steam in the condensation chamber is at saturation with a pressure of 30 mbar. The steam passes through an insulated hose and enters the chamber, in which the sample is mounted and cooled by a recirculating chiller. Flow rate of the chiller is monitored by a flowmeter (SITRANS FM MAG5000 and SITRANS FM MAG 1100, SIEMENS). In the chamber, the steam pressure and temperature, as well as the sample surface temperature, are continuously measured at 2 Hz. All other sensors of the system are connected to the same data acquisition device and measured at 2 Hz (Beckhoff). Excess condensate is collected at the storage. At the exit of the chamber, a vacuum pump (RC 6, VACUUBRAND) is used to drive a stable steam flow.

A schematic of the chamber is found in **Supplementary Figure 3**.

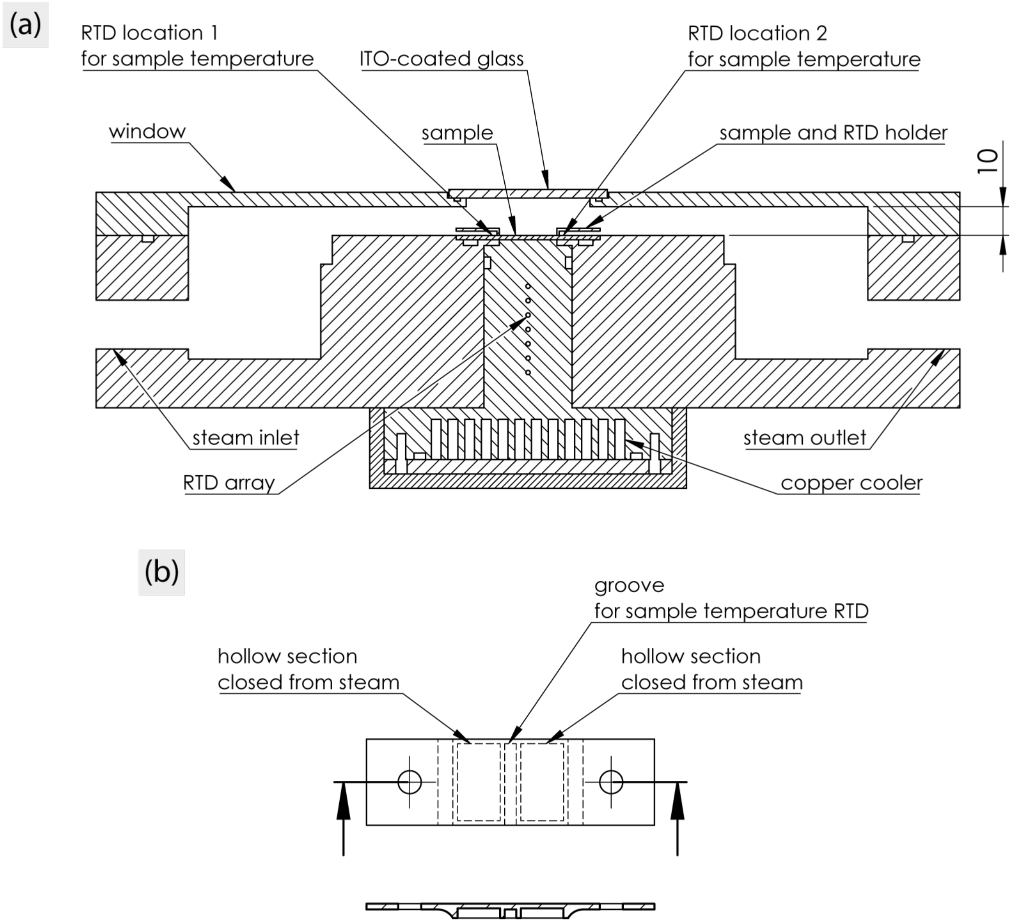

Supplementary Figure 3: Top-view cross section of the condensation chamber. Dimension in mm. Reproduced from our previous work[2] which is under the Creative Commons licence CC BY-NC-ND 4.0.

The condensation chamber is where the sample is mounted and tested. The base of the chamber is milled from a block of polyether ether ketone (PEEK), and its front window is milled from a block of poly(methyl methacrylate) (PMMA).

Refer to **Supplementary Figure 3a**. The sample of size 50 mm × 20 mm is mounted vertically in the middle of the chamber, on one end of the copper cooler, which measures 20 mm × 20 mm in size. During operation, only the centre 20 mm × 20 mm region of the sample directly on top of the cooler is exposed to steam, and the remaining two 15 mm × 20 mm side regions of the sample are insulated from direct steam exposure. The insulation is achieved by the design

of the mounts (**Supplementary Figure 3b**). On top of each side region, a 3D-printed hollow polycarbonate mount is screwed into the chamber to provide pressure onto the sample and fix it onto the cooler. Below each mount, a groove is present to accommodate and fix a Pt 1000 Class A resistance temperature detector (RTD) (P1K0.516.1K.A.152.D.S, IST), which is attached to the sample with Kapton tape for measuring the surface temperature. The mean of the two surface temperature RTDs is taken as the measured value. For superhydrophobic boehmite, the absolute discrepancy of each RTD from the mean is continuously monitored to be ≈ 0.25 K, which is exceptionally close to the inherent uncertainty of these RTDs (0.2 K). Together, the 2 redundant RTDs help (1) confirm that the temperature is not imbalanced across the surface due to imperfect contact of the sample with the cooler; and (2) exclude the possibility of one of the sensors being incorrectly attached or become accidentally detached during an experiment.

The accurate measurement of the surface temperature is critical to the reliability of heat transfer coefficient determination. To this end, the type of temperature sensor and its method of attachment to the surface are carefully chosen. RTDs are selected which are generally more accurate than thermocouples. In particular, the thin-film RTDs in our experiments are with a flat construction and designed for surface temperature measurements. The side for temperature measurement is ensured to maintain direct and constant contact with the surface. First, before and after each experiment, the Kapton tape and the RTD are confirmed to have attached tightly to the surface with no visible looseness or displacement upon nudging. Second, the RTDs are clamped by the 3D-printed polycarbonate mount. The pressure applied by these mounts ensure an even more secure placement of the RTDs and their continuous and constant contact with the surface. On the other hand, the hollow structure of these mounts provide further thermal insulation of the RTD from the saturated steam, in addition to the thermal insulation provided

by the Kapton tape itself. Lastly, the placement of our RTDs do not interfere with condensation and droplet dynamics. This is because of the fact that the region they are attached to the sample is not directly exposed to steam (**Supplementary Figure 3a**) and therefore condensation on the sensors is negligible. Active condensation only occurs in the observed 20 mm × 20 mm area, which is directly exposed to the steam.

For steam conditions in the chamber, the steam pressure is measured with a capacitance gauge (CMR 362, Pfeiffer Vacuum) and the steam temperature is measured with two Pt 1000 Class A RTDs (P1K0.516.1K.A.152.D.S, IST). Similarly, the mean of the two steam temperature RTDs is taken as the measured value.

The sample is fixed by the mounts onto the front end of the cooler. Between the sample and the cooler, a thermal paste (KP 99, Kerafol) is applied. The cooler is milled from a block of copper (CW004A, Durofer). The back end of the cooler is a heat exchanger with a coolant recirculated by a chiller (WKL 2200, LAUDA). Between the front and back ends of the cooler, an array of 7 Pt 100 Class A RTDs (Thermo Sensor) is used to determine the temperature gradient and the heat flux using the thermal conductivity of the cooler (394 W m^{-1} K^{-1}). For this part of the cooler where the 7 RTDs are located, there is a thin closed air gap between the cooler and the PEEK chamber base as extra insulation (not drawn in **Supplementary Figure 3a**).

The temperature of the back end of the cooler is directly controlled by the temperature of the coolant, which is input to the chiller. Given a temperature difference between the steam (fixed at saturation temperature, 24.1 ˚C) and the back end of the cooler (varied between 20 and -10 ˚C), the tested condensing surface determines its resulting subcooling and the heat flux through

it. Better-performing surfaces result in a smaller range of resulted subcooling, as less thermal resistance is present between the surface and the steam.

At the centre of the PMMA window, an indium-tin-oxide-coated borosilicate glass (Diamond Coatings) is installed into a cut-out. A slight voltage is applied to the coating to provide minimal heating to remove condensate fogging as necessary.

A flow condensation environment is employed for several reasons. First, it is more relevant to realistic condenser conditions in applications. Second, microscopic observation of saturated steam condensation (rarely performed in existing literature) requires a close placement of the objective from the condensing surface (therefore a ≈ 10 mm window-surface distance, **Supplementary Figure 3a**). In such a confined space, a stagnant vapour environment may not provide the ideal reservoir conditions of saturated steam for condensation to occur. Also, if steam flow was not actively maintained as in the current configuration, the intensive condensation of saturated steam would itself drive an uncontrolled strong vapour flow in the ≈ 10 mm gap due to the rapid phase change from vapour to liquid. Therefore, the well-controlled steam flow environment in this work ensures that condensation is not limited by the supply of saturated steam, and that all surfaces are tested under a similar flow field. Third, the continuous introduction of steam removes any accumulating non-condensable gases, which are already minimal based on leakage tests, for more reliable heat transfer coefficient measurements. Lastly, roaming events occur in all directions and are not affected by the flow. All samples are tested under the same controlled environment with the same steam conditions.

Observation setup

The observation setup is similar to our previous work,[3] consisting of a light source, a beam splitter, a microscope objective, and a high-speed camera. In front of each, the light source and the high-speed camera, there is a set of lenses. Refer to **Figure 1b** in the main text.

A microscope objective (UPlanFl 4x/0.13 PhL, Olympus) is placed in front of the chamber window, focused on the condensing surface. White light is generated by an LED (LEDD1B and MCWHF2, Thorlabs) and carried through an optical fibre (core size 1 mm, QP1000-2-UV-BX, Ocean Optics) to the optical assembly.

The first set of lenses, in front of the light source, comprises of a collimator followed by a convex lens. The convex lens is used to adjust the size of the illuminated area on the sample. If no convex lens was installed, the collimated light would be reflected by the beam splitter and enter the microscope objective to be refocused to an illuminated area of 1 mm in diameter (core size of the optical fibre) on the condensing surface. Instead, in our setup, the convex lens placed after the collimator refracts the collimated light, resulting in a variable diameter of the final illuminated area on the sample. In our work, the diameter is ≈ 4 mm, which coincides with the field of view of the microscope objective, i.e. the entire field of view is illuminated.

The reflected light from the sample passes through the microscope objective and the beam splitter to another set of lenses, in front of the high-speed camera (FASTCAM SA1.1, Photron), to focus onto the image sensor. As the microscope objective is focused on the condensing surface, the light from the surface is focused at infinity after exiting the objective. A macro lens (AF Micro-Nikkor 200 mm f/4D IF-ED, Nikon) set to infinity focus is used to refocus the

image onto the sensor. A frame rate of 10000 fps can be achieved for a reflective surface such as boehmite.

Experimental procedures

We follow similar experimental procedures to our previous work.[1,2] For each experiment, the chamber is first pumped at least overnight by the vacuum pump with an ultimate pressure of 0.002 mbar. The bypass valve parallel to the steam flowmeter and the bypass valve parallel to the regulating and metering valves are open, and the 3-way steam control valve before the steam flowmeter is closed. The components between the steam control valve and the vacuum pump are thus pumped. Pumping overnight allows the removal of all condensate from previous experiments remaining in these components. The chamber then reaches its minimum pressure, which is below our measurement limit of 0.01 mbar.

After one night, the chamber is vented, and the tested sample is mounted onto the cooler with thermal paste as described above. The chamber is pumped down again, and the pump runs continuously from this point to the end of the experiment.

The chiller is set to a temperature of 25 ˚C, slightly higher than the target saturation temperature of 24.1 ˚C, such that when steam is later introduced into the chamber, condensation does not immediately occur. Flow rate of the coolant is set to 180 ± 10 L h^{-1}. The boiler is filled with deionised water, set to be open to atmosphere using the 3-way steam control valve, and turned on. The water is boiled at > 1.4 bar for 30 min to degas. At the same time, the two bypass valves are cycled open-and-close multiple times to release the trapped gases in them.

After 30 min of degassing, the two bypass valves are closed and the steam control valve is switched from the atmospheric side to the chamber side. Steam is introduced into the chamber. The LED light source is turned on and set to maximum until the end of the experiment. Using the regulating and metering valves, the steam pressure in the chamber is slowly increased to

the target pressure of 30 mbar. At the same time, boiler power is adjusted to maintain a pressure of 1.4 ± 0.01 bar until the end of the experiment. As the chamber pressure approaches 30 mbar, the chiller is set to the first set point of 20 ˚C. Condensation takes place as the surface temperature reaches below the chamber steam temperature. At this stage, the steam in the chamber has already reached saturation. Fluctuations in the measured chamber steam temperature can be seen to closely follow the fluctuations in the measured chamber steam pressure.

When the chiller reaches 20 ˚C, the flow rate is readjusted to 180 ± 10 L h^{-1} to compensate for the change caused by thermal contraction of the coolant. The system is then adjusted to stabilise by fine-tuning the boiler power and the metering valve, until it can maintain steady-state conditions without intervention.

The glass window is then checked for condensate fogging. If present, a voltage of 10 V is applied to the indium-tin-oxide coating at a current of ≈ 580 mA for 15 s to generate minimal heat to remove the condensate fog on the window. No measurable increase in chamber steam temperature by this heating is detected. The microscope objective is refocused. The system is then left without intervention for 1 min, and the measurements within this minute are used to compute the steady-state data point (subcooling, heat flux and heat transfer coefficient) at this set point. During this minute, the chamber pressure has to maintain at 30 ± 0.5 mbar and the boiler pressure has to maintain at 1.4 ± 0.01 bar without intervention. Approximately 30 s into this minute, a video is taken with the high-speed camera at 10000 fps for ≈ 1 s, which is the condensation behaviour attributed to this data point. The procedure in this paragraph is repeated when more than one video is desired for this chiller set point.

The chiller is then set to proceed to the next set point. For the results presented in **Figure 3**, a total of 7 set points are set, from 20 to -10 ˚C at 5 ˚C intervals. As the chiller reaches the next set point, the procedures above are repeated, i.e. coolant flow rate readjustment, system stabilisation, condensate fogging check, refocusing, steady-state measurement and high-speed video recording.

After the data point at the last chiller set point (-10 ˚C) is complete, the chiller is set to 25 ˚C. The boiler is turned off, but steam continuously passes over the sample as there is still a pressure difference between the boiler and the pump, preventing the surface from reaching freezing temperatures as the chiller takes time to heat up.

The LED light source is turned off. When the coolant temperature reaches 5 ˚C, the chiller is turned off. The 3-way steam control valve from the boiler, and the shutoff valve to the pump, are closed, stopping the steam flow. The chamber is vented, and the sample is removed from the cooler and blown dry with a weak nitrogen flow.

Computation of heat fluxes and heat transfer coefficients

Heat flux is computed from the linear temperature gradient measured by the RTD array in the cooler (**Supplementary Figure 3a**). The one-dimensional Fourier's law of conduction states:

$$q = kA\frac{dT}{dx}$$

where k = 394 W m^{-1} K^{-1} is the thermal conductivity of the cooler, A is the cross-sectional area of the cooler, and dT/dx is the temperature gradient obtained from the linear fit of the 7 measured temperatures by the array. q is the heat flow through the cooler. As the cooler is insulated with a closed air gap and a thermally insulative PEEK chamber base, the heat flow through the cooler can be assumed to be equal to the heat flow through the condensing surface, i.e. there are negligible thermal losses. Therefore, the heat flux of the condensing surface q'' can be computed by dividing the heat flow q by its area, $q'' = q/A$, where A = 20 mm × 20 mm = 4 × 10^{-4} m^2 is the area of the condensing surface instead.

The heat transfer coefficient h is defined as:

$$h = \frac{q''}{\Delta T}$$

where $\Delta T = T_{\text{steam}} - T_{\text{surf}}$ is the subcooling, i.e. the temperature difference between the measured steam temperature T_{steam} and the measured surface temperature T_{surf}.

Every data point represents steady-state measurements of a 1-min period. In **Figure 3a** and **Supplementary Figures 4**, **6**, **18**, **19**, **20** and **24**, for each surface, 7 data points are plotted, reporting the heat transfer coefficients and heat fluxes for 7 subcooling. The temporal mean of the measured subcooling in this 1-min period (2 Hz, 120 instantaneous values) is the reported subcooling for this data point. Similarly, the reported heat flux and heat transfer coefficient for this data point is the temporal mean of the respective values in this 1-min period.

<u>Goodness of fit for heat flux measurements</u>

The linear fit for heat flux is excellent, with the coefficient of determination R^2 very close to 1 throughout the experiment. Refer to **Supplementary Figure 4**. The crosses and error bars refer to the mean heat flux measurements for superhydrophobic boehmite at steady state as reported in **Supplementary Figure 18**. The tiny individual dots refer to the instantaneous heat flux measurements, each with $R^2 > 0.98$. As seen, we not only obtain a good fit at steady state conditions, but also when we transition from one steady state to another (dots between the steady state crosses).

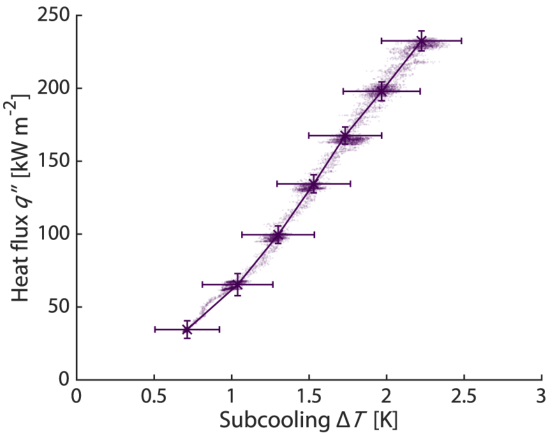

Supplementary Figure 4: Heat fluxes q'' for superhydrophobic boehmite. Crosses refer to measurements at steady state. Each circular dot refers to an instantaneous heat flux measurement with $R^2 > 0.98$, indicating the goodness of fit throughout the experiment. Source data are provided as a Source Data file.

Uncertainty propagation

Error bars reported in **Figure 3a** and **Supplementary Figures 4**, **6**, **18**, **19**, **20** and **24** are calculated from both the uncertainty of the sensors as well as the fluctuations of the measurements. We follow the procedures for uncertainty propagation,[4] similar to our previous work.[2] A summary is provided below.

Each RTD for temperature measurement has an inherent uncertainty of $\delta_{\mathrm{RTD}} = 0.2$ K. The steam temperature is measured from the mean of 2 RTDs. Its uncertainty can be written as:

$$\delta_{T_{\mathrm{steam}}} = \frac{1}{2}\sqrt{\delta_{\mathrm{RTD}}^2 + \delta_{\mathrm{RTD}}^2}$$

Similarly, the surface temperature is measured from the mean of 2 RTDs. Its uncertainty can be written as:

$$\delta_{T_{\mathrm{surf}}} = \frac{1}{2}\sqrt{\delta_{\mathrm{RTD}}^2 + \delta_{\mathrm{RTD}}^2}$$

The uncertainty in subcooling is then:

$$\delta_{\Delta T} = \sqrt{\delta_{T_{\mathrm{steam}}}^2 + \delta_{T_{\mathrm{surf}}}^2}$$

The heat flux is obtained from the linear fitting of 7 RTDs. The uncertainty in linear fitting can be written as:

$$\delta_{\mathrm{fit}} = \delta_{\mathrm{RTD}}\sqrt{\frac{N_{\mathrm{RTD}}}{N_{\mathrm{RTD}}\sum x_{\mathrm{RTD}}^2 - (\sum x_{\mathrm{RTD}})^2}}$$

Here, $N_{\mathrm{RTD}} = 7$ is the number of RTDs in the array for heat flux measurement, and x_{RTD} is their individual location. δ_{RTD} is then used to calculate the uncertainty in heat flux, as follows:

$$\delta_{q''} = k\frac{A_{\mathrm{cooler}}}{A_{\mathrm{cond}}}\delta_{\mathrm{fit}}$$

The array of RTDs is located within the cooler. k is the thermal conductivity of the cooler and A_{cooler} is the cross-sectional area of the cooler. A_{cond} is the condensing area.

The uncertainty in the heat transfer coefficient is computed from the uncertainties in heat flux, surface temperature and steam temperature above. It can be written as:

$$\delta_h = \sqrt{\left[\frac{1}{\Delta T}\delta_{q''}\right]^2 + \left[\frac{-q''}{(\Delta T)^2}\delta_{T_{\text{steam}}}\right]^2 + \left[\frac{q''}{(\Delta T)^2}\delta_{T_{\text{surf}}}\right]^2}$$

The steady state mean values of ΔT and q'' are used to compute the uncertainty in h at the corresponding steady state.

The uncertainties above are calculated from the inherent uncertainty of the RTDs. For the error bars, the fluctuations in instantaneous measurements during the steady state are also included. For simplicity, we sum the uncertainties calculated above and the standard deviation of the instantaneous measurements, and report the sum as the error bars.

Validation with the Nusselt model

The measurements for filmwise condensation are validated with the Nusselt model. For condensation from saturated steam on a small vertical plate, the model predicts the heat transfer coefficient to be:[5]

$$h = 1.13\left[\frac{\rho(\rho - \rho_{\text{steam}})gh_{fg}k^3}{L\mu(T_{\text{steam}} - T_{\text{surf}})}\right]^{\frac{1}{4}}$$

where h_{fg} is the latent heat of vaporisation at 30 mbar (= 2443.9 kJ kg^{-1}). L is the length of the vertical condensing area in the gravitational direction (= 20 mm). ρ_{steam} is the density of the steam (= 0.021904 kg m^{-3}). ρ, k and μ are the density, thermal conductivity and dynamic viscosity of the condensate respectively, evaluated at the condensate film temperature $T_{\text{film}} = (T_{\text{steam}} + T_{\text{surf}})/2 = T_{\text{steam}} - \Delta T/2$ of the subcooling. For T_{steam} = 24.1 ˚C (saturation temperature at 30 mbar) and 0.5 K ≤ ΔT ≤ 12 K, the range of values at T_{film} are:

$$23.829\ ^\circ\text{C} \geq T_{\text{film}} \geq 18.079\ ^\circ\text{C}$$

$$997.30\ \text{kg m}^{-3} \leq \rho \leq 998.54\ \text{kg m}^{-3}$$

$$0.60453\ \text{W m}^{-1}\ \text{K}^{-1} \geq k \geq 0.59450\ \text{W m}^{-1}\ \text{K}^{-1}$$

$$9.1431 \times 10^{-4}\ \text{kg m}^{-1}\ \text{s}^{-1} \leq \mu \leq 1.0506 \times 10^{-3}\ \text{kg m}^{-1}\ \text{s}^{-1}$$

Figure 3a plots the prediction with the Nusselt model onto the heat transfer measurements on superhydrophobic and pristine boehmite. The measurements on pristine boehmite, on which filmwise condensation occurs, match closely with the predictions of the model. Therefore, our experimental setup and procedures are validated to accurately measure heat transfer coefficients.

Accessible range of subcooling

The accessible range of subcooling is dependent on the tested surface. When a surface is tested, its temperature (thus the subcooling) cannot be directly imposed. We vary a thermal boundary condition of the system instead and measure the resultant surface temperature and subcooling. This concept can be visualised with the schematic in **Supplementary Figure 5**. Our experimental setup is shown at the top, with the temperature gradient in the middle and the thermal resistance circuit at the bottom. The boundary conditions are the cooler back end temperature $T_{\mathrm{coolerback}}$ and the steam temperature T_{steam}. Thermal resistances come from the cooler copper cylinder, thermal paste, sample thickness, and the condensation.

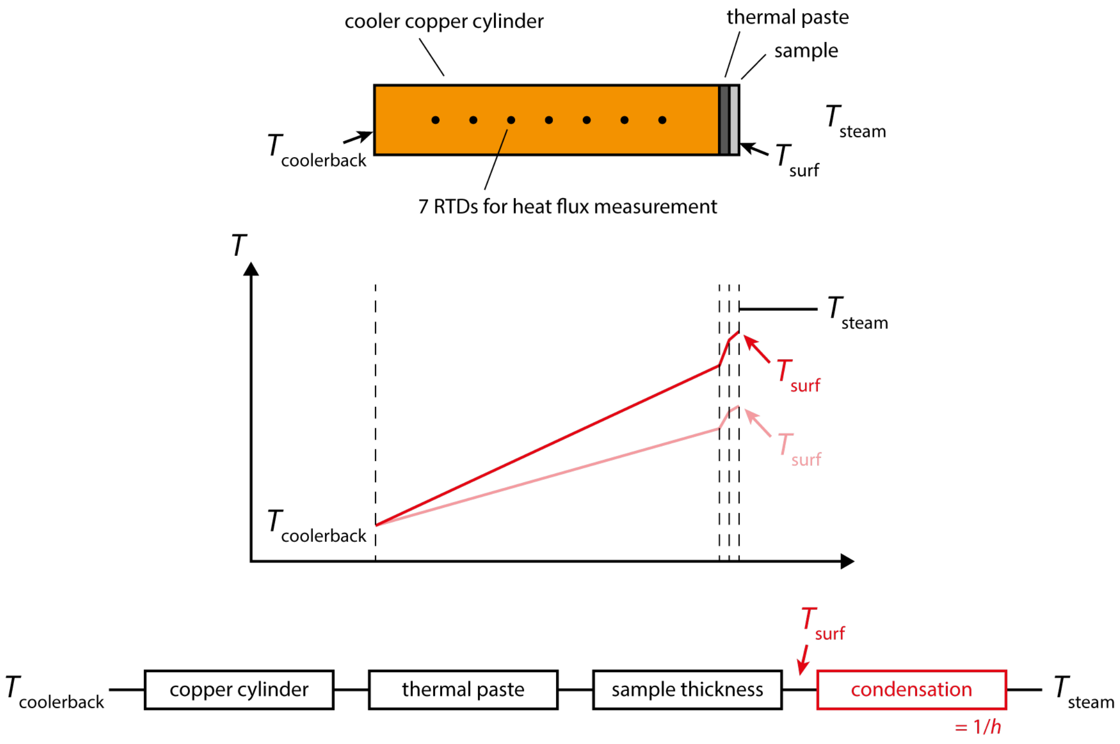

Supplementary Figure 5: Experimental setup (top), temperature gradient (middle), and the thermal resistance circuit (bottom).

In **Figure 3a**, we did not deliberately choose to plot filmwise condensation up to a subcooling of 10 K: It is a direct consequence of a sample surface with lower heat transfer coefficients. In

Supplementary Figure 5, for given cooler back end and steam temperatures, the surface temperature T_{surf} (and the subcooling) is determined by the relative magnitude of thermal resistances composing the circuit. When we test different samples, the condensation thermal resistance (= $1/h$) is different. Therefore, the condensation efficiency of the sample surface directly determines the subcooling achieved.

The steam temperature is fixed (saturation temperature at 30 mbar, 24.1 ˚C). To reach different subcooling levels, we impose the cooler back end temperature at 7 points from 20 to -10 ˚C (see above). For the lowest set cooler back end temperature (-10 ˚C), the subcooling reaches 2.2 K on the superhydrophobic boehmite and 10.1 K on the pristine boehmite. This is a direct result of the higher heat transfer coefficient of the superhydrophobic boehmite: Because it has a lower condensation heat transfer resistance (= $1/h$), the surface temperature is closer to the steam temperature, resulting in a lower subcooling. In **Supplementary Figure 6**, we plot the subcooling and heat transfer coefficient of the two surfaces against the cooler back end temperature.

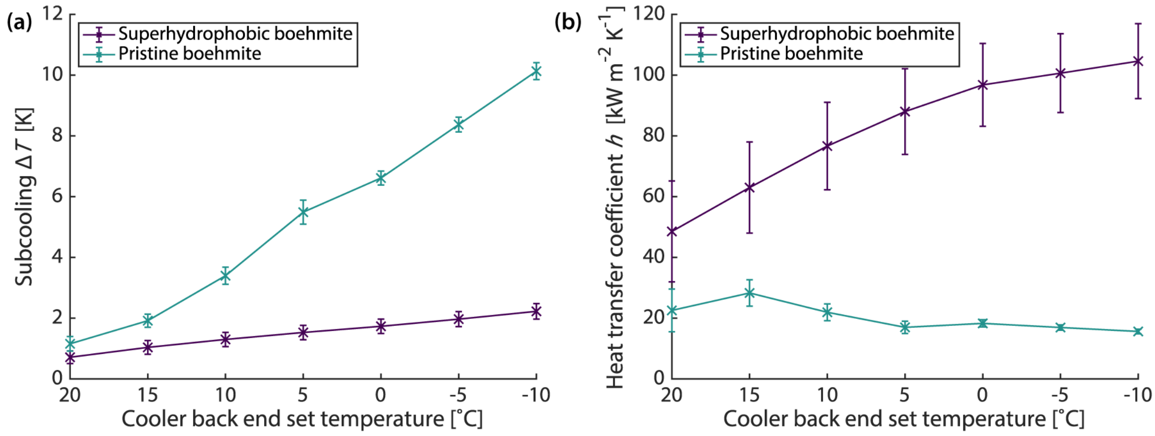

Supplementary Figure 6: Subcooling and heat transfer coefficients at the 7 cooler back end temperatures. Source data are provided as a Source Data file.

S3. Effect of microstructures on droplet motion

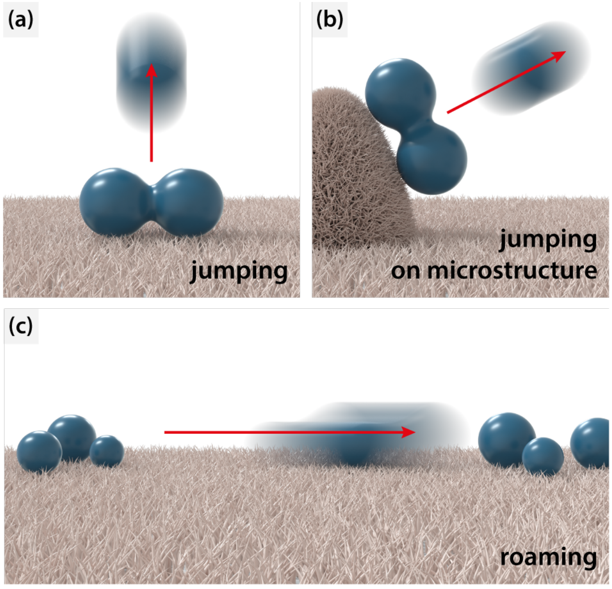

Supplementary Figure 7: Different droplet motion behaviour. (a) Motion from droplet jumping is normal to the surface. (b) When there are microstructures which are at the same length scale as the droplets, the direction of jumping can be altered. (c) Roaming can occur without microstructures. **a** and **c** have appeared in the 2023 American Physical Society Division of Fluid Dynamics Gallery of Fluid Motion on https://doi.org/10.1103/APS.DFD.2023.GFM.V0073 which is under the Creative Commons licence CC BY-NC 4.0.

Coalescence-induced jumping of microdroplets is normal to the surface (**Supplementary Figure 7a**). Therefore, when it occurs on the lateral walls of microstructures, the resultant jumping motion would be sideways,[6] as shown in **Supplementary Figure 7b**.

The mechanism for roaming motion is entirely different, in that it can occur on solely nanostructured surfaces, producing a planar motion without the presence of any microstructures (**Supplementary Figure 7c**). The planar (tangential) motion is inherent to the phenomenon itself.

Hierarchical condensation

On hierarchical surfaces, which possess nanostructures overlayed onto microstructures, hierarchical condensation may occur as described in Ref.[7]. It is a phenomenon where small condensate droplets form within the microcavities below larger droplets suspended on top of these cavities (**Supplementary Figure 8**). For favourable microcavity morphologies, the small droplets can navigate out of the cavity towards the larger droplet. Upon coalescence with the larger droplet, also known as the sink, the small droplet appears to be “absorbed” into it. New condensate droplets can then form within the emptied cavity, and the cycle repeats.[7]

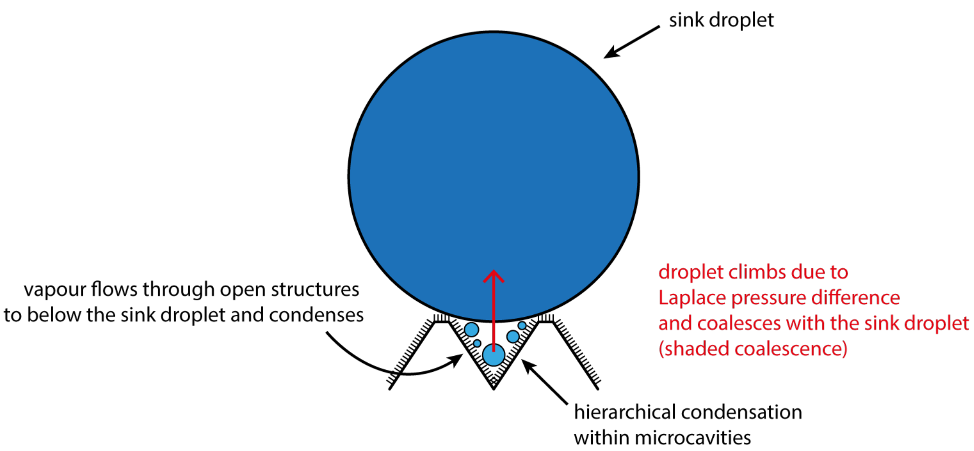

Supplementary Figure 8: Hierarchical condensation as described in Ref.[7].

Hierarchical condensation cannot occur on boehmite or other solely nanostructured surfaces. In hierarchical condensation, the larger droplets are suspended over large microstructures (≈ 30 – 40 µm in pitch in Ref.[7]). In comparison, droplets on boehmite surfaces are suspended over features with characteristic sizes of ≈ 20 – 40 nm, 3 orders of magnitude lower. As a result, the presence of a large number of droplets below a suspended droplet depicted in **Supplementary Figure 8** and Ref.[7] is not possible on solely nanostructured surfaces for hierarchical condensation. The nanostructure cavities can only accommodate at most a few nuclei at reasonable subcooling (**Supplementary Figure 9**).

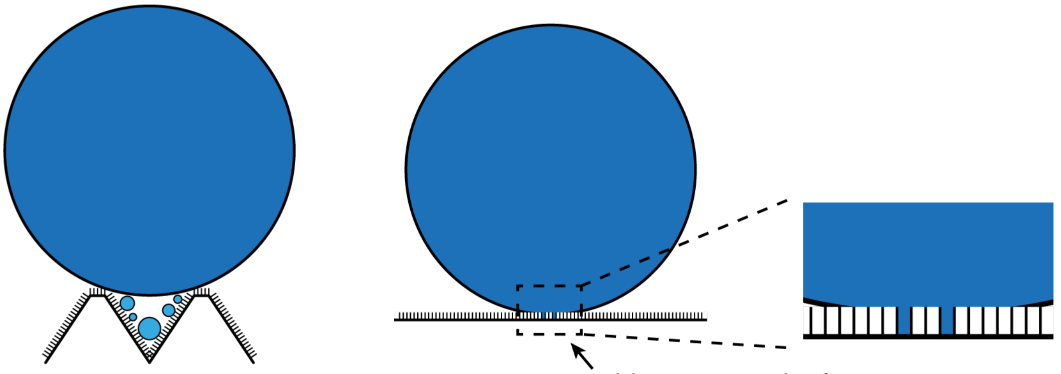

Supplementary Figure 9: Difference in structure sizes and condensation within the cavities.

Moreover, hierarchical condensation requires the constant inflow of vapour into the structure cavities so that condensate can form below the suspended droplets.[7] This is only possible with open structure cavities, where there are wide paths to allow the flow of vapour. Nanostructures are typically too dense and thin for vapour to flow through. For boehmite and similar nanostructures, the cavities are also closed on the sides by nanowalls. Therefore, they can only be wetted when directly exposed to the vapour from their top opening, that is, without the presence of a droplet at this location blocking the vapour access (**Supplementary Figure 8** and left of **Supplementary Figure 9**). There is no other straightforward flow path for vapour to enter below the droplet suspended on top of these structures blocking the top opening.

In summary, hierarchical condensation cannot occur on boehmite or other solely nanostructured surfaces, and thus cannot be responsible for roaming motion on our surfaces.

It can further be shown that even if there is hierarchical condensation, it is not possible to induce roaming. Firstly, hierarchical condensation on solely nanostructured surfaces would mean wetted nanocavities. In **Supplementary Figure 8**, hierarchical condensation results in condensate within the microstructures in the nano-Cassie state, suspended on top of

nanostructures with high mobility.[7] The micro- and nanostructure cavities are not wetted. However, on solely nanostructured surfaces, the nanocavities do not have another tier of roughness. Condensation within them can result in truly wetted cavities, and dewetting them requires more (and external) work, which is provided by the coalescence of participating droplets during roaming motion. Before roaming, these cavities remain wetted, resulting in stable partial-Wenzel droplets above as we describe in the main text.

Secondly, the coalescence of two liquid bodies with a vast size mismatch cannot produce enough momentum required for roaming. From the hydrodynamic perspective, the smaller body is reduced to becoming a small perturbation to the larger droplet. This perturbation is viscously dissipated without material effect to the coalesced bulk. From the energetic perspective, up to ≈ 20% of surface energy is released[8] upon coalescence when there is no size mismatch between two spherical droplets ($M = (r_2 - r_1)/(r_1 + r_2) = 0$, r_1 and r_2 are the radii of a smaller and larger droplet respectively). When there is an order of magnitude size difference[7] (e.g. 10 μm vs. 100 μm), $M = 0.818$ and only 0.9% of surface energy is released. On solely nanostructured surfaces, the difference is even larger and the released surface energy becomes negligible. The calculations are shown below.

Consider two droplets of radii r_1 and $r_2 > r_1$. They coalesce on an ideal non-wetting surface such that they can be considered spherical. The total volume V_{12} is conserved during coalescence:

$$V_{12} = \frac{4}{3}\pi r_1^3 + \frac{4}{3}\pi r_2^3 = \frac{4}{3}\pi r_{12}^3$$

where r_{12} is the radius of the droplet after coalescence. Then,

$$r_1^3 + r_2^3 = r_{12}^3$$

For $M = (r_2 - r_1)/(r_1 + r_2)$ defined as the size mismatch (equivalent to the definition based on diameters in **Supplementary Information S12**),

$$r_2 = r_1 \frac{1+M}{1-M}$$

Substituting the equations, we have the following expressions for r_1^2 and r_2^2:

$$r_1^2 = r_{12}^2 \left[1 + \left(\frac{1+M}{1-M}\right)^3\right]^{-2/3}$$

$$r_2^2 = r_{12}^2 \left[1 + \left(\frac{1+M}{1-M}\right)^{-3}\right]^{-2/3}$$

The surface energy is the product between the surface area and surface tension γ. The total surface energy of the two droplets before coalescence is

$$(4\pi r_1^2 + 4\pi r_2^2)\gamma$$

and the surface energy of the droplet after coalescence is

$$4\pi r_{12}^2 \gamma$$

The excess surface energy E_{ex} that is released from coalescence, normalised by the surface energy before coalescence, is

$$E_{\mathrm{ex}} = 1 - \frac{4\pi r_{12}^2 \gamma}{(4\pi r_1^2 + 4\pi r_2^2)\gamma} = 1 - \left\{\left[1 + \left(\frac{1+M}{1-M}\right)^3\right]^{-2/3} + \left[1 + \left(\frac{1+M}{1-M}\right)^{-3}\right]^{-2/3}\right\}^{-1}$$

In **Supplementary Figure 10**, E_{ex} is plotted for $0 \leq M < 1$, where E_{ex} approaches 0 as M approaches 1.

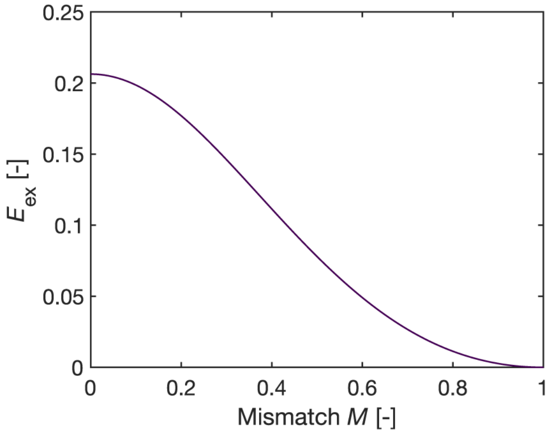

Supplementary Figure 10: Normalised excess surface energy E_{ex} for different mismatch M.

In conclusion, not only hierarchical condensation, as described in Ref.[7], cannot occur on solely nanostructured surfaces, but even if it were to occur (hypothetically), it would not provide a viable pathway for momentum generation. Roaming cannot result from hierarchical condensation.

S4. Image processing, droplet measurement and event tracking

High-speed videos in the form of image sequences are first processed in Adobe Lightroom Classic to enhance contrast. The resulting image sequence is imported into ImageJ (National Institutes of Health) for further processing. The measurement of participating droplets and the main droplet are used to study the evolution and mechanics of roaming events.

Participating droplets

Participating droplets refer to all the droplets which coalesce in a roaming event. Before coalescence, they possess a spherical cap geometry. Using the oval tool in ImageJ, the centre and diameter of the circular participating droplets are measured. They are measured at the frame (time) before the beginning of their coalescence, determined by a visible perturbation to their interface. Therefore, a time is assigned to every participating droplet. The time of the first participating droplet is set to $t = 0$ and the time of every other is set relative to this first droplet. The duration of the roaming event is the duration between the first and the last participating droplet coalescence.

For participating droplets much smaller than the main droplet, their coalescence with the main droplet cannot be seen as they may lie under the main droplet at the point of coalescence. However, their coalescence with the main droplet can be confirmed by the absence of these participating droplets after the main droplet traverses and leaves behind a clear renewed area. In these cases, the participating droplets are measured at the frame when they are last seen.

The directionality of roaming events can be seen in the spatial distribution of participating droplets. If a convex hull is drawn around the participating droplets, we observe a lower circularity ($= 4\pi(\mathrm{area}/\mathrm{perimeter}^2)$) of the hull for roaming events (0.70 ± 0.08) than the hull for clustered multi-droplet coalescence (0.89 ± 0.06).

Main droplet

The main droplet refers to the traversing droplet, resulted from the coalescence of previous participating droplets, which sweeps and coalesces with the remaining participating droplets until termination of the roaming event. The main droplet has an irregular geometry. The contour of their projected shape on the condensing surface is measured using the polygon tool in ImageJ. The centroid of the measured polygon is the location of the main droplet at that time.

We begin to measure the main droplet when its contour can be clearly seen for the first time, and measure the main droplet for each frame afterwards. For most roaming events, the main droplet comes to rest on the surface at the end of the event. In this case, the main droplet is measured until 20 frames (2 ms) after the time of the last participating droplet. Occasionally, the main droplet jumps and departs from the surface to terminate the roaming event. In this case, the main droplet is measured until 1 frame (0.1 ms) after the time of the last participating droplet.

Main droplet contours measured in ImageJ are imported into MATLAB for processing using code on the MATLAB Central File Exchange.[9]

Additional notes on measurement

The measurement of participating droplets and the main droplet are used to study the evolution and mechanics of roaming events. Therefore, longer events which proceed in all in-plane directions are chosen for measurement. We also avoid measuring roaming events which are blocked by a substantially larger droplet (compared to the size of the main droplet before coalescence with it), which brings the event into an abrupt stop as the tangential momentum is largely dissipated after the main droplet coalesces into it. Every measured event begins spontaneously, i.e. there is no incoming landing droplet to transfer momentum, and all participating droplets are at rest before the beginning of the event without remnant oscillations from previous droplet motion events.

It is noted that there are inherent difficulties in the extraction of the behaviour of the main droplet. Discontinuities arise as it traverses and coalesces with participating droplets. For example, at the moment of coalescence with a participating droplet, the main droplet instantaneously gains in projected area, resulting in a sudden movement in the location of its centroid and the velocity of the main droplet cannot be well defined. Similarly, jumps occur in the main droplet mass as participating droplets coalesce into the main droplet. Discontinuities in the velocity and mass prohibit reliable computation of the momentum and kinetic energy of the main droplet.

S5. Roaming on various nanostructured surfaces

Roaming has been reported on superhydrophobic silicon nanograss (black silicon)[10] and copper nanowires[11] in the literature. We demonstrate its generality on different nanostructures with observation on superhydrophobic titanium dioxide nanorods[12] and copper(II) hydroxide nanoneedles[13] in this section. It is noted that due to the poor reflectivity of these samples, the image quality is significantly lower than boehmite in **Figure 3b**. Nevertheless, condensation modes can still be identified.

Titanium dioxide nanorods

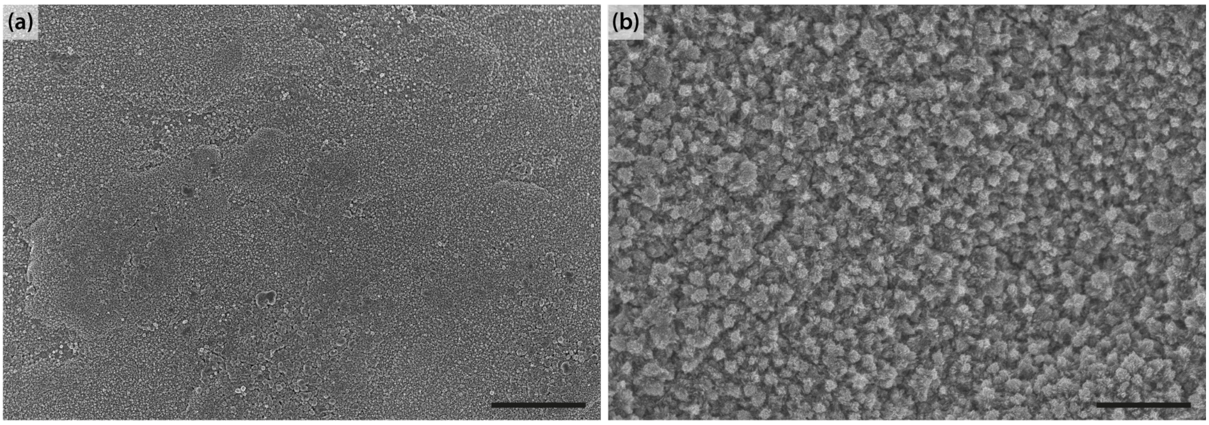

Supplementary Figure 11: SEM images of titanium dioxide nanorods with the silane coating at different magnifications. (a) No prominent structures are present at the microscale. Scale bar: 20 μm. (b) Structures at the nanoscale. Scale bar: 2 μm.

Branched nanorod structures are prepared on flat titanium substrates. These structures are nano-hierarchical, i.e. both the larger-scale and smaller-scale structures are at the nanoscale. No structures are in the microscale to affect jumping direction as in **Supplementary Figure 7b** found on conventional micro-nano hierarchical surfaces. The structured surface is then functionalised with 1H,1H,2H,2H-perfluorodecyltrichlorosilane. Fabrication procedures can be found in our recent work.[12] In the following, we expose samples with branch lengths of ≈ 90 nm (type b-NR-90 as specified in the work[12]) to the same condensation conditions as boehmite. SEM images of the structures can be found in **Supplementary Figure 11**.

These surfaces are superhydrophobic with a measured advancing contact angle of 159.5° ± 1.3° and contact angle hysteresis of 1.8°. Roaming is seen when subcooling is increased. See **Supplementary Figure 12**.

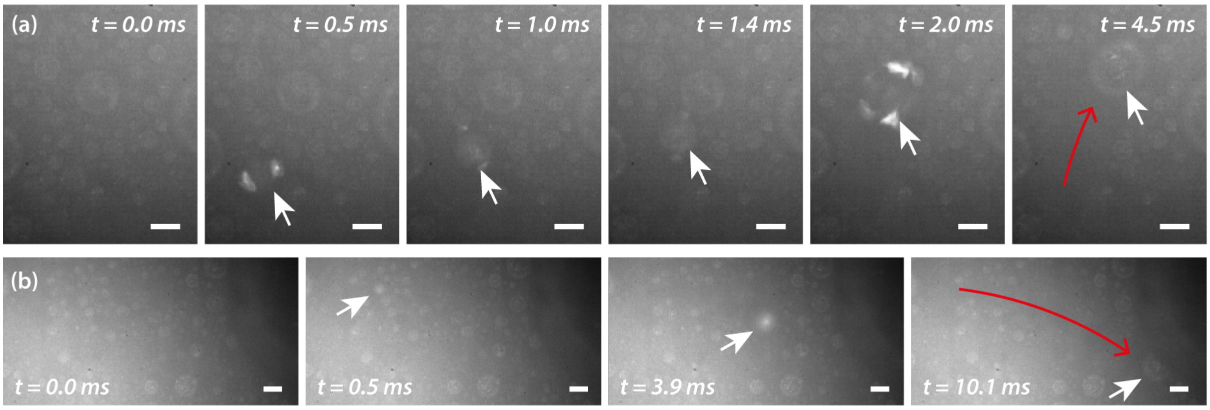

Supplementary Figure 12: Roaming on superhydrophobic titanium dioxide nanorods. White arrow points to the main droplet. Red arrow indicates approximate trajectory. After roaming, there are no droplets left on the surface along the trajectory. (a) Upward event against gravity. Subcooling is at 2.7 K. Scale bars: 100 µm. (b) Rightward event. Subcooling is at 1.7 K. Scale bars: 100 µm.

Copper(II) hydroxide nanoneedles

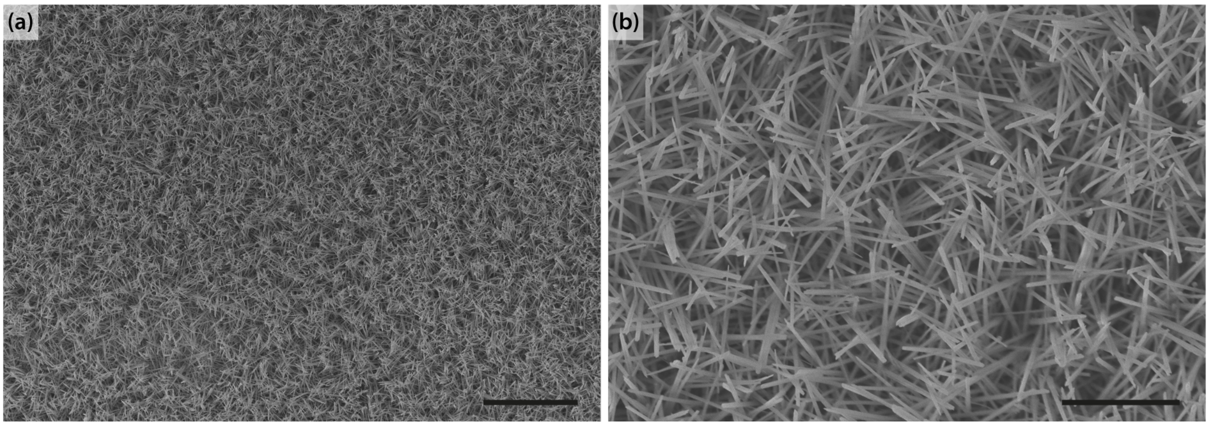

Supplementary Figure 13: SEM images of copper(II) hydroxide nanoneedles with the pPFDA coating at different magnifications. (a) No prominent structures are present at the microscale. Scale bar: 20 µm. (b) Structures at the nanoscale. Scale bar: 5 µm.

Nanoneedle structures are prepared on flat copper substrates according to procedures from our recent work,[13] and then functionalised with pPFDA using iCVD (**Methods**). Similarly, there are no structures at the microscale. SEM images of the structures can be found in **Supplementary Figure 13**. We expose these surfaces to the same condensation conditions as boehmite.

These surfaces are superhydrophobic with a measured advancing contact angle of 160.2° ± 1.3° and contact angle hysteresis of ≈ 0°. Roaming is seen when subcooling is increased. See **Supplementary Figure 14**.

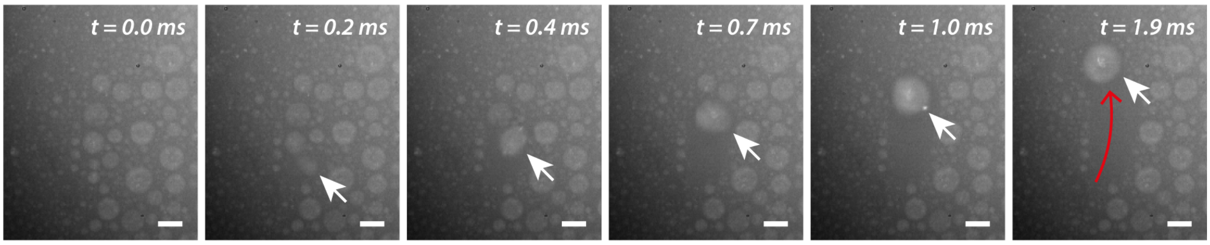

Supplementary Figure 14: Upward roaming on superhydrophobic copper(II) hydroxide nanoneedles against gravity. White arrow points to the main droplet. Red arrow indicates approximate trajectory. Subcooling is at 0.7 K. Scale bars: 100 µm.

S6. Evolution of roaming events

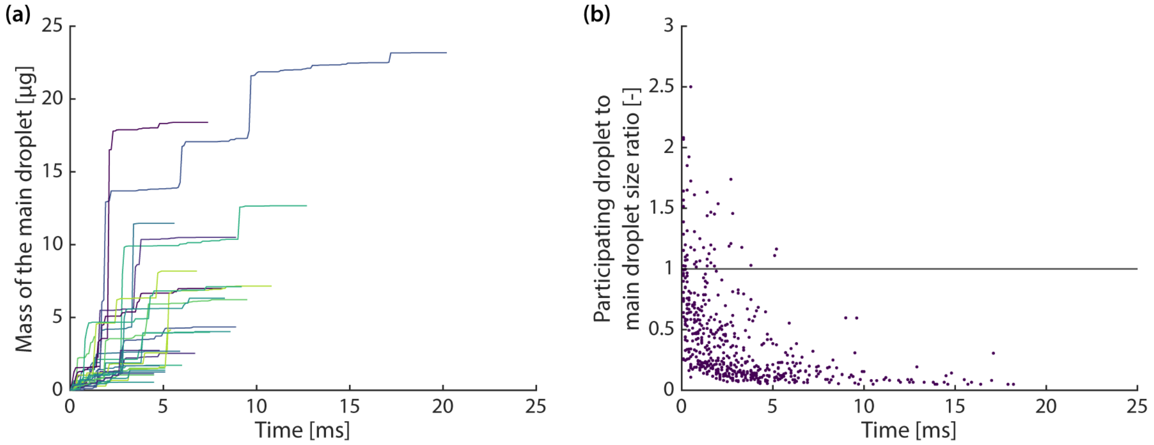

Supplementary Figure 15: (a) Mass of the main droplet over time. (b) Ratio of participating droplet sizes relative to the main droplet size, from all measured roaming events. Source data are provided as a Source Data file.

Assuming all droplets exhibit a spherical cap shape and a constant contact angle (= 162.0°) with the surface, the volume of each participating droplet can be estimated from the measured diameter. The volume of the main droplet at any given time can then be estimated by summing the volume of all participating droplets which have coalesced at this time. The mass of the main droplet is computed by multiplying the volume with the density. **Supplementary Figure 15a** shows the increase in the mass of the main droplet over time for different roaming events. The mass gain of each event varies significantly.

In a roaming event, as the main droplet gains in size, the size of participating droplets relative to it reduces. In **Supplementary Figure 15b** we compute the ratio of participating droplet sizes relative to the main droplet size. The ratio is computed as follows: (1) The volume of the main droplet at each time point is converted to an equivalent diameter as if it assumed a spherical cap shape and a contact angle of 162.0°; (2) For each time point, if there is at least one

participating droplet coalescing, the ratio at this time point is computed by dividing the diameter of the largest participating droplet coalescing, by the equivalent diameter of the main droplet. It can be seen in **Supplementary Figure 15b** that this ratio drops below unity at ≈ 5 ms.

S7. Real roaming velocity

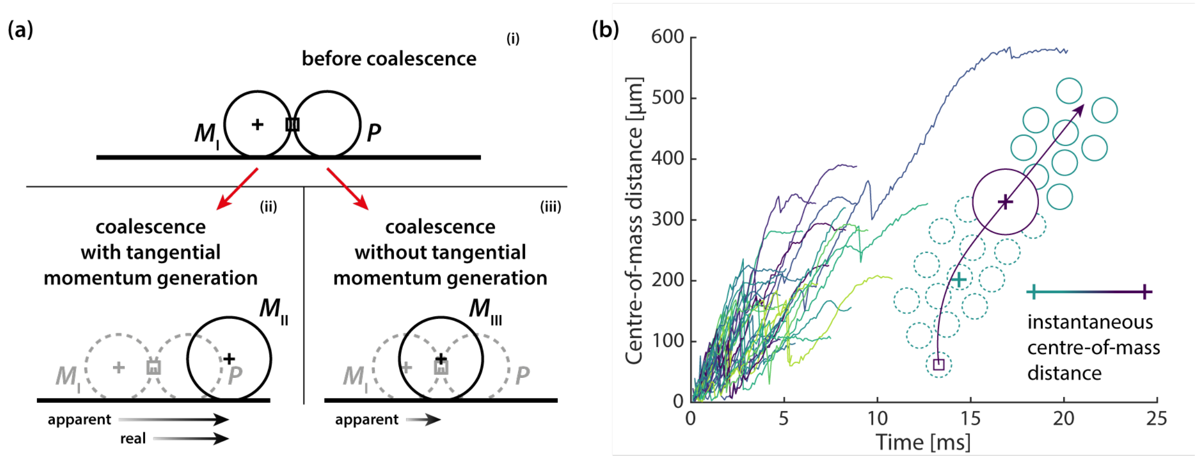

Supplementary Figure 16: (a) Shift in the centre of mass of the main droplet upon coalescence, with and without tangential momentum generation. Cross: Centre of mass of the main droplet. Square: Centre of mass of the system of droplets before the coalescence (M_{I} and P). (b) Evolution of the distance between the centre of mass of the main droplet and the centre of mass of the system of participating droplets for the measured roaming events. Schematic illustrates a roaming event in progress. Square: Starting location of the roaming event. Violet circle: Main droplet. Turquoise circles: Coalesced (dashed) and to-be-coalesced (solid) participating droplets. Violet cross: Centre of mass of the main droplet. Turquoise cross: Centre of mass of the system of coalesced participating droplets (dashed turquoise circles). Source data are provided as a Source Data file.

Supplementary Figure 16a illustrates the shift in the centre of mass of the main droplet upon coalescence. The original location of the main droplet M_{I} and a participating droplet P is shown in Panel (i). Upon coalescence, two outcomes can occur for the location of the new main droplet, M_{II} in Panel (ii) or M_{III} in Panel (iii). If there is tangential momentum generation (Panel (ii)), the location of the new main droplet M_{II} will be away from the centre of mass of the system of droplets before the coalescence (M_{I} and P), indicated by the square. In contrast, if there is no tangential momentum generation (Panel (iii)), the location of the new main droplet M_{III} will be unshifted from, and coincide with, the centre of mass of the system of M_{I} and P.

However, even if there is no tangential momentum generation (Panel (iii)), it is clear that there is a displacement of the location of the main droplet from M_{I} to M_{III}, due to the addition of the mass of P to M_{I}, away from the location of M_{I}. This displacement is apparent, and not a result of tangential momentum generation. Tracking the location of the main droplet results in the apparent roaming velocity as described in the main text (**Figure 2c**).

To isolate the result of tangential momentum generation, we compute the distance between the current centre of mass of the main droplet at every time instant of a roaming event and the centre of mass of the system of participating droplets which have coalesced by that instant. See the schematic in **Supplementary Figure 16b**. A roaming event in progress is illustrated. The distance between the centre of mass of the main droplet (violet cross) and the centre of mass of the system of participating droplets which have coalesced (turquoise cross) is measured over time of the roaming event. The measurements of several roaming events are individually plotted in **Supplementary Figure 16b** with different colours. The main droplet is consistently "ahead" of the system of coalesced participating droplets at any given time. An increase in the distance over time indicates a continuous tangential momentum generation in one in-plane direction.

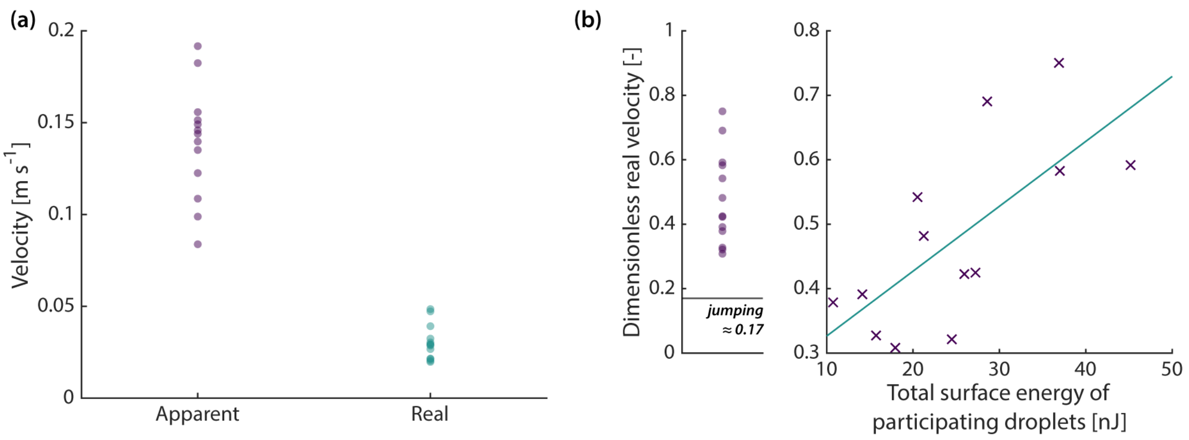

Supplementary Figure 17: (a) Apparent roaming velocity and real roaming velocity at 5 ms. (b) Left: Distribution of the dimensionless real velocity of roaming. For jumping, the dimensionless velocity is 0.17.[8] Right: Dimensionless real velocity against the total surface energy of participating droplets. Source data are provided as a Source Data file.

To extract the characteristic real roaming velocity as a result of tangential momentum generation, we consider the centre-of-mass distance gained at 5 ms, after which the intensity of coalescence is reduced, while viscous dissipation and gravity become important (see main text). A comparison of the apparent and real roaming velocities for the same set of roaming events is shown in **Supplementary Figure 17a**. Next, we divide the real roaming velocities by the theoretical maximum, which is as if all excess liquid-vapour interfacial energy were converted to in-plane translational kinetic energy of the main droplet. The result is a dimensionless real velocity of 0.48 ± 0.14, compared to 0.17 of droplet jumping from binary coalescence.[8] See the left plot of **Supplementary Figure 17b**. Although the efficiency of jumping might be higher from the coalescence of more droplets, this is very limited to cases where the droplets prior to coalescence are so closely packed that they are all almost in direct contact.[14] Such closely packed arrangement is extremely rare in reality. The efficiency rapidly deteriorates when the droplets are just slightly apart.[14]

Finally, we plot the dimensionless real velocity against the total liquid-vapour interfacial surface energy of coalesced participating droplets at 5 ms for each event, in the right plot of **Supplementary Figure 17b**. An increasing trend is observed, suggesting that smaller droplets may benefit the energy conversion.

S8. Additional heat transfer measurements

Heat flux against subcooling on boehmite

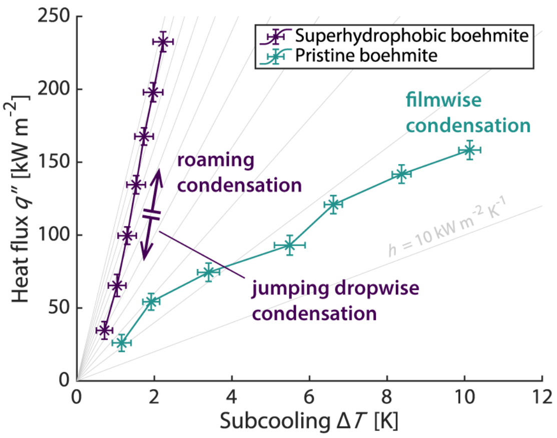

Supplementary Figure 18: Measured heat fluxes q'' at steady state. Lines of constant heat transfer coefficient h are shown in grey, from 10 to 110 kW m^{-2} K^{-1} at intervals of 10. $dh/d(\Delta T) > 0$ when $dq''/d(\Delta T) > h$. On the superhydrophobic surface, two modes of condensation are observed. Source data are provided as a Source Data file.

Supplementary Figure 18 plots the heat flux against subcooling for the superhydrophobic boehmite and pristine boehmite surfaces. The superhydrophobic surface always requires smaller subcooling to sustain the same heat flux. **Figure 3a** plots the heat transfer coefficient against subcooling. On the superhydrophobic surface, roaming condensation (mean = 183.2 kW m^{-2}, last 4 points from the left in **Supplementary Figure 18**) sustains a 175% higher heat flux than jumping dropwise condensation (mean = 66.5 kW m^{-2}, first 3 points from the left in **Supplementary Figure 18**), owing to the combined effect of a higher thermal driving force, i.e. subcooling, and a higher heat transfer coefficient.

Heat transfer coefficient and subcooling

The heat transfer coefficient is always a manifestation of the current mode of condensation and condensate dynamics. When the subcooling is varied, the mode and/ or dynamics may change, leading to changes in heat transfer, which is reflected in the calculated heat transfer coefficient.

There exist thus some general trends and their corresponding physical explanation is based on changes in mode and/ or dynamics. (1) When the condensate assumes the form of a film (filmwise condensation), the surface is covered by the condensate and further direct condensation takes place on this continuous film during heat transfer. The heat transfer coefficient decreases when subcooling is increased, due to the thickening of the condensate film. (2) When the condensate assumes the form of discrete droplets (this includes purely gravitational dropwise condensation, jumping dropwise condensation and roaming condensation), during heat transfer, direct condensation occurs on the condensate droplets. The droplet distribution is evolving continuously and is sensitive to nucleation rates and droplet diameter, which are in turn controlled by the subcooling. Smaller droplets pose lower thermal resistances and heat is preferentially transferred through them. Therefore, the heat transfer coefficient increases when subcooling is increased, due to the lowering of nucleation diameter and higher nucleation rates. However, this relies on the premise that the increased nucleation rates and lower nucleation diameters are sustainable, that is, there is continuously an abundance of these smaller droplets over time. The (increased) rate of condensate generation thus has to be balanced by the rate of removal to avoid accumulation. Only then the condensation mode and condensate dynamics are stable, which result in stable heat transfer coefficients, at this subcooling.

The increase in heat transfer coefficient with subcooling has been observed on lubricant-infused surfaces[2] as well as surfaces coated with polymer brushes.[15] Condensate remains in the form of droplets on them in the subcooling range considered. As an example, **Supplementary Figure 19** overlays the heat transfer coefficient measurements for a surface composed of polydimethylsiloxane (PDMS) brushes under the same experimental conditions in the same experimental setup[15] on the plot in **Figure 3a**. **Supplementary Figure 20** plots the heat transfer coefficients against heat flux. Due to their lower hydrophobicity than superhydrophobic surfaces, the lack of droplet self-propulsion (jumping or roaming) leads to lower heat transfer coefficients.

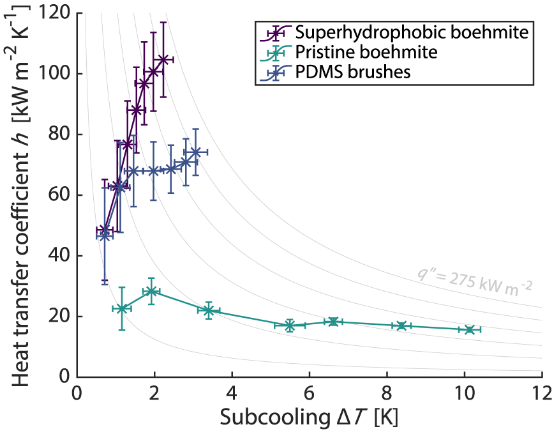

Supplementary Figure 19: Heat transfer measurements for a surface composed of PDMS brushes[15] compared to superhydrophobic and pristine boehmite from **Figure 3a**. Data for PDMS brushes from Ref.[15]. Source data are provided as a Source Data file.

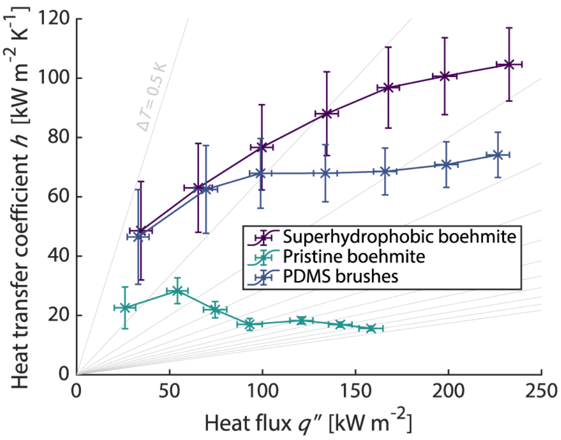

Supplementary Figure 20: Heat transfer measurements in **Supplementary Figure 19**, with heat flux on the x-axis. Data for PDMS brushes from Ref.[15]. Lines of constant subcooling ΔT are shown in grey, from 0.5 to 11.5 K at intervals of 1. Source data are provided as a Source Data file.

For the trend of increasing heat transfer coefficient with increasing subcooling to break down on the abovementioned surfaces, subcooling has to be increased to the point that the available sites for nucleation on the surface become saturated.

For superhydrophobic surfaces, there is the additional element of surface structures. Spontaneous droplet motion on top of these structures result in lower droplet diameters at a given subcooling, resulting in higher heat transfer coefficients than a flat surface. The increasing heat transfer coefficient trend with increasing subcooling holds as long as the condensate does not nucleate and accumulate within the structure cavities. When the subcooling is high enough for nucleation to occur within the cavities, in addition to the increased nucleation rate from the increase in subcooling, there is an increase in the area available for nucleation, from the cavity walls. For condensate not to accumulate within the structures, there must exist effective droplet removal mechanisms. In our work, we show that roaming, which naturally occurs on superhydrophobic surfaces at these high subcooling levels, efficiently employs excess surface energy from coalescence to drive cavity dewetting.

Roaming is a stable mode of condensation in which the condensate does not accumulate over time. In other words, the higher nucleation rate and the smaller nucleation diameters are both sustainable at the elevated subcooling levels where roaming is dominant. The increasing trend of heat transfer coefficient with subcooling is further extended through the transition to roaming condensation.

S9. Rates of condensate volume removal and surface area renewal

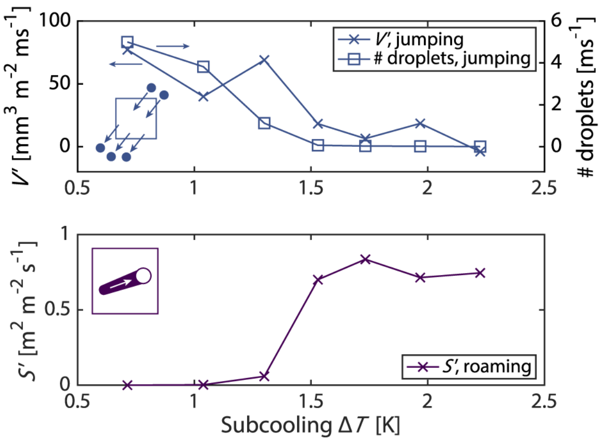

Supplementary Figure 21: Condensate volume removal rate V' from jumping, calculated from the difference between the volume of jumped droplets entering and exiting the crop region. Unit: mm^3 of condensate per m^2 of condensing surface per millisecond (top, left axis). Total number of jumped droplets entering and exiting the crop region. Unit: Number per millisecond (top, right axis). Surface area renewal rate S' from roaming. Unit: m^2 of surface area renewed per m^2 of condensing surface per second (bottom). At ≈ 1.5 K, a transition is seen from the reduction of V' and number of droplets from jumping; and the increase in S' from roaming. Source data are provided as a Source Data file.

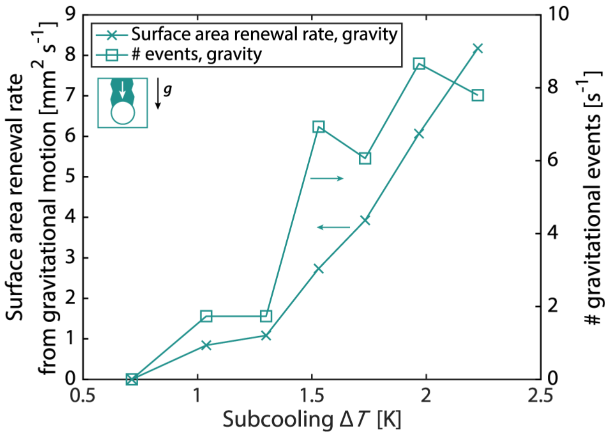

Supplementary Figure 22: Surface area renewal rate from gravitational motion of droplets. Unit: mm^2 of surface area renewed per second (left axis). Number of gravitational motion events observed. Unit: Number per second (right axis). At ≈ 1.5 K, a sharp transition in slope in both measurements is seen. Source data are provided as a Source Data file.

Condensation exhibits a transition as subcooling increases. Refer to **Supplementary Figure 21**. At the transition subcooling of 1.5 K, the condensate volume removal rate V' and the number of droplets from jumping decreases and approaches zero, whereas the surface area renewal rate S' from roaming increases sharply from near zero. Note that S' includes only the area renewed by roaming motion.

The transition to roaming significantly increases the surface area renewal rate from gravitational motion as well. Refer to **Supplementary Figure 22**. Crosses and the left axis show the surface area renewal rate from gravitational motion. Squares and the right axis show the number of observed gravitational events. At the transition subcooling of 1.5 K when roaming emerges, there is a sharp change in slope in both measurements. While the motion of roaming itself renews over 70% of the condensing area every second as indicated by S', it also accelerates the gravitational departure of droplets and the surface area renewal rate from such departure. This is because roaming events often terminate with a droplet at rest on the surface, which markedly increases the growth rate of droplets on the surface: The serial motion in roaming intensifies coalescence frequencies. Droplets then attain the critical size for gravitational departure much earlier than through only the direct condensation of vapour at a site, or local coalescence of sessile droplets in place, without roaming involved.

Measurement procedures for the quantities above are detailed in the following.

To avoid edge effects, we choose to consider a square crop region of size 2250 μm × 2250 μm at the centre of our field of view with a size approximately of 3160 μm × 3160 μm. This field of view is approximately at the centre of the condensing surface of size 20000 μm × 20000 μm. This section provides the details for the measurement of condensate volume removal rate and the number of droplets from jumping, and the surface area renewal rates from roaming.

Condensate volume removal rate and the number of droplets from jumping

The condensate volume removal rate from jumping is measured at different subcooling with ImageJ. As droplets jump from the condensing surface, they are affected by the steam flow and gravity. Therefore, numerous droplets that have jumped away from the surface travel across the field of view and the crop region. To identify the volume of the droplets that jumped from the surface within the crop region, we measure the total volume of jumped droplets entering the crop region (inflow) and the total volume of jumped droplets exiting the crop region (outflow). The difference between outflow and inflow is the condensate volume removal rate from jumping in the crop region.

For the lowest 3 subcooling, 50 ms of video is measured as jumping is the dominant mode. For the highest 4 subcooling, 1000 ms of video is measured as jumping vanishes. When a jumped droplet crosses the boundary of the crop region, it is measured as a circle with the oval tool of ImageJ. The measured diameter is converted to the volume of the jumped droplet as $(4/3)\pi(d/2)^3$. The total volumetric inflow and outflow, and their difference, in the timeframe considered can then be obtained. In **Supplementary Figure 21**, we report the normalised condensate volume removal rate from jumping per area of condensing surface per time. Negative values are possible when in the timeframe considered, more droplets land and come to rest in the crop region than those which jump from the condensing surface. We also report

in **Supplementary Figure 21** the total number of jumped droplets entering and exiting the crop region.

The above volumetric method is adopted because jumping is dominant when subcooling is low. Droplets on the surface are small and the surface area renewed from jumping cannot be easily discerned. The condensate volume removal rate provides an estimate for the renewal of the surface. Lastly, it is noted that roaming can terminate in jumping as well around the transition subcooling. The reported removal rate includes all jumping droplets.

In the measurement for V', very small droplets are ignored. Although jumped droplets are out of plane, they are only slightly out of focus and the sideway steam flow carries the droplets across the measurement boundaries before they could jump very far away from the surface or focal plane, resulting in small uncertainties. The clear downward trend in V' and the number of jumped droplets measured, as subcooling increases, indicates the cessation of droplet jumping consistent with other results.

Surface area renewal rate from roaming

The surface area renewal rate from roaming is measured at different subcooling with ImageJ. Roaming motion exhibits clear directionality compared to local multi-droplet coalescence. For each subcooling, 11546 frames are examined for roaming motion events (recorded at 10000 fps, thus duration approximately 1.15 s). At approximately the end of each event (i.e. the main droplet has either stopped or jumped), the polygon tool in ImageJ is used to enclose and measure the renewed surface area, indicated by the lack of condensate droplets and higher reflectivity. Only the renewed surface area within the crop region is measured. A roaming event is only considered when it begins within the field of view, at least one participating droplet is

in the crop region, and not initiated by a landing droplet visibly transferring momentum. In **Figure 3c** and **Supplementary Figure 21**, we report the normalised surface area renewal rate from roaming per area of condensing surface per time.

Surface area renewal rate from gravitational motion

The surface area renewal rate from gravitational motion is measured at different subcooling with ImageJ. An event is considered when it is clearly gravitational, that is, it is in the direction of gravity and the size of the droplet in concern is much larger than the surrounding ones. For each subcooling, 11546 frames are examined for gravitational motion events (recorded at 10000 fps, thus duration approximately 1.15 s). The polygon tool in ImageJ is used to enclose and measure the renewed surface area, indicated by the lack of condensate droplets and higher reflectivity. Only the renewed surface area within the crop region is measured. A gravitational event does not have to begin within the field of view to be considered, but it cannot be seen to have obtained its motion inertia from a landing droplet. In **Supplementary Figure 22**, we report the surface area renewal rate from gravitational motion per time, and the number of observed gravitational events per time, in the crop region. Note that for gravitational motion, these measurements cannot be directly normalised to the entire condensing surface as their values depend on the location of the field of view. However, the important trend over subcooling is maintained.

S10. Critical nucleation diameter and transition subcooling

From classical nucleation theory, the diameter at which nucleation occurs, i.e. the critical nucleation diameter d_{crit}, follows the expression:[16]

$$d_{\mathrm{crit}} = \frac{4\sigma}{(RT_{\mathrm{surf}}/v_{\mathrm{l}})\ln[P_{\mathrm{v}}/P_{\mathrm{sat}}(T_{\mathrm{surf}})] - P_{\mathrm{v}} + P_{\mathrm{sat}}(T_{\mathrm{surf}})}$$

where σ is the surface tension, R is the ideal gas constant, v_{l} is the specific volume of the condensate, P_{v} is the steam pressure and $P_{\mathrm{sat}}(T_{\mathrm{surf}})$ is the saturation pressure at the surface temperature. **Figure 3c** plots the variation of the critical nucleation diameter d_{crit} with subcooling for 30 mbar saturated steam, as well as the cavity size distribution of boehmite.

Transition subcooling

Condensation transitions from the jumping dropwise mode to the roaming mode upon increase in subcooling past the transition threshold. This is observed on boehmite (main text) and other types of nanostructures (**Supplementary Information S5**). On titanium dioxide nanorods, the transition subcooling is similar to boehmite (≈ 1.5 K), due to a similar density of nanostructures. However, when the nanostructures are sparser resulting in larger cavities on copper(II) hydroxide nanoneedles, the transition subcooling is notably reduced to 0.7 K.

Supplementary Figure 23 compares the structures of boehmite and copper(II) hydroxide. The density of nanostructures on copper(II) hydroxide is much lower with significantly larger cavities.

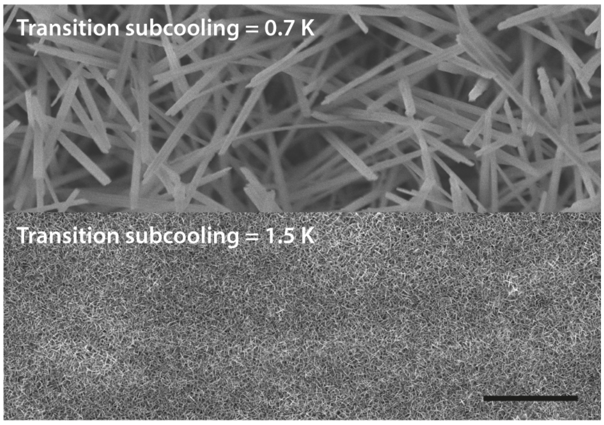

Supplementary Figure 23: SEM images of copper(II) hydroxide nanoneedles (top) and boehmite nanowalls (middle), at the same magnification. Both are coated with pPFDA. Scale bar: 2 μm.

As we increase the subcooling on superhydrophobic copper(II) hydroxide nanoneedles, a transition from jumping dropwise condensation to roaming condensation is observed. These structures flood as we further increase the subcooling. **Supplementary Figure 24** shows the heat fluxes q'' and heat transfer coefficients h at different subcooling.

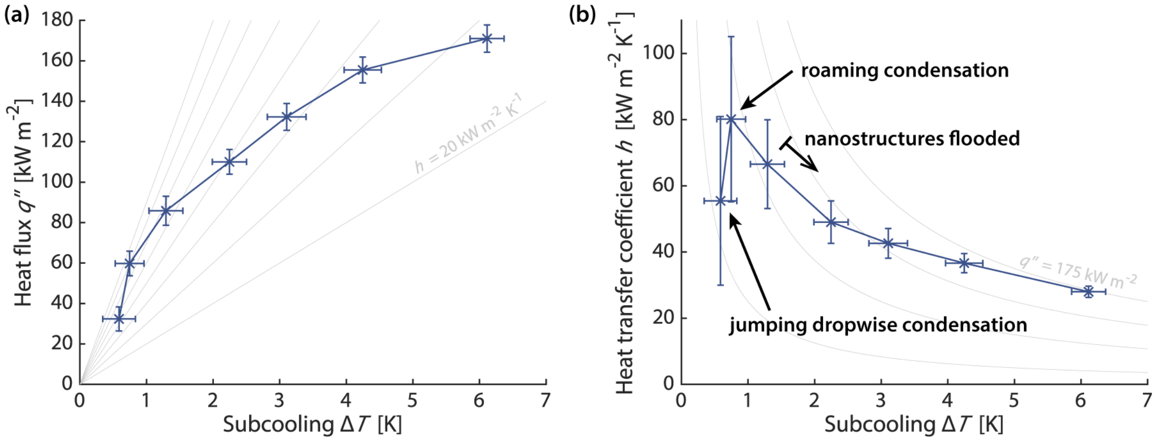

Supplementary Figure 24: Heat transfer at different subcooling on superhydrophobic copper(II) hydroxide nanoneedles. (a) Measured heat fluxes q'' at steady state. Lines of constant heat transfer coefficient h are shown in grey, from 20 to 90 kW m^{-2} K^{-1} at intervals of 10. (b) Heat transfer coefficients h corresponding to **a**. Lines of constant heat flux q'' are shown in grey, from 25 to 175 kW m^{-2} at intervals of 50. Source data are provided as a Source Data file.

At the lowest subcooling measured (0.6 K), condensation is predominantly jumping dropwise. At a higher subcooling of 0.7 K, the condensation mode quickly transitions to roaming. At this point, we measure the highest heat transfer coefficient. However, as subcooling is further increased, the surface floods, accompanied by a gradual decrease in h. **Supplementary Figure 25** shows the condensation behaviour at different subcooling.

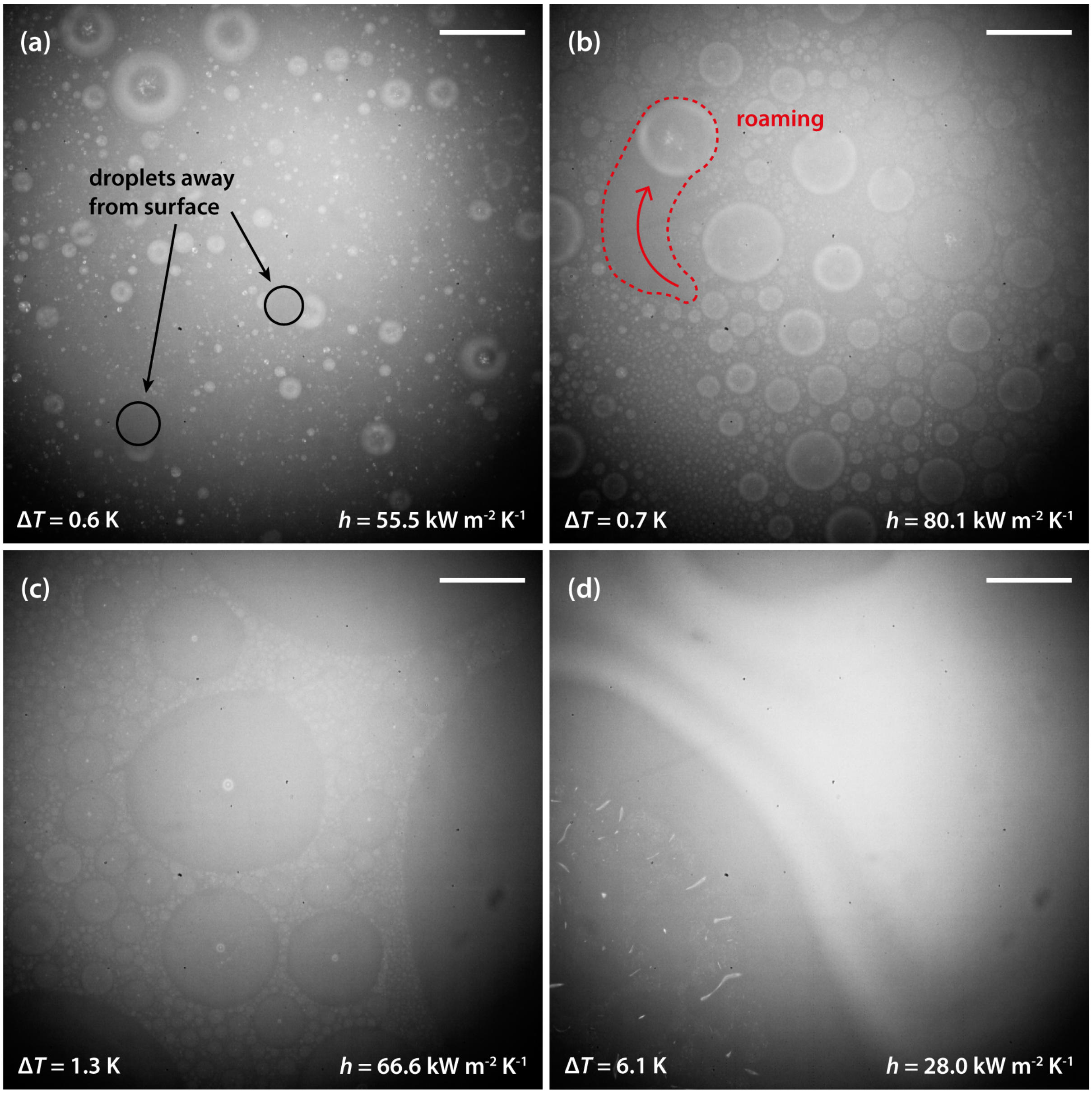

Supplementary Figure 25: Snapshots of condensation behaviour at different subcooling on superhydrophobic copper(II) hydroxide nanoneedles. Jumping dropwise condensation is seen in **a**, where the droplets which have jumped away from the surface block the droplets on the surface from view. Transition is seen from (a) jumping dropwise to (b) roaming condensation, and finally to (c) and (d) flooded condensation. Scale bars: 500 µm.

Control and promotion of roaming

Roaming condensation naturally occurs with its high heat transfer efficiency when the subcooling is in the required range. Therefore, if this range is located at higher subcooling levels, roaming can occur at these higher subcooling levels as well. This in turn enables higher heat transfer coefficients at higher subcooling levels, maximising efficiency. Based on our understanding developed in this work (main text and above), the location of this subcooling range (which begins at the transition subcooling) depends on the morphology of the nanostructures. As roaming emerges when the critical nucleation diameter becomes smaller than the nanostructure cavities, reducing cavity size moves the transition subcooling and roaming subcooling regime to higher levels of subcooling. On the other hand, it may also be advantageous to have nanostructures with optimised geometry[17] for enhanced flooding resistance on the surface structure level, so that synergistically, the higher end of the range can be further extended to even higher subcooling levels. **Supplementary Figure 26** summarises how roaming should be controlled and promoted.

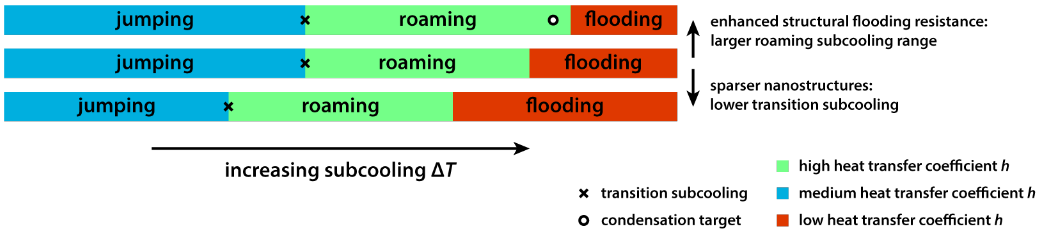

Supplementary Figure 26: Control and promotion of roaming. Sparser nanostructures lower the transition subcooling and roaming occurs at lower subcooling levels. Enhanced flooding resistance on the surface structure level extends the subcooling range to higher levels. Heat transfer efficiency is maximised when roaming occurs at high subcooling levels, which should be targeted, as shown for example in the top bar of the figure.

S11. Volumetric nucleation rate and cavity filling timescale

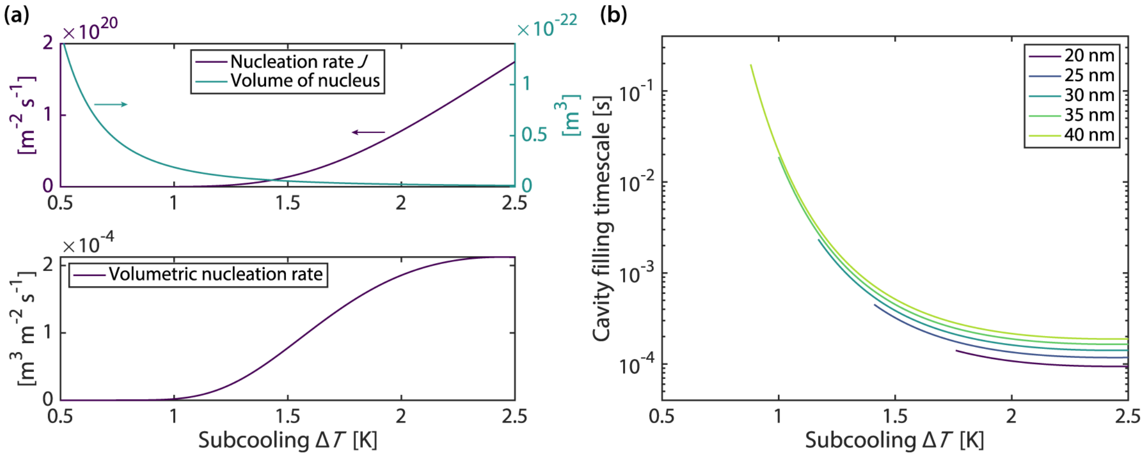

Supplementary Figure 27: (a) Nucleation rate J and volume of each nucleus V_{nucl} (top), and their product, the volumetric nucleation rate (bottom). Transition can be seen at ≈ 1.5 K. (b) Timescale to fill the nanostructure cavities of sizes 20 – 40 nm. For each size, there is a minimum subcooling to nucleate within it, determined by the critical nucleation diameter.

Volumetric nucleation rate

We extend our model to include the dynamics of nucleation in **Supplementary Figure 27**. We first calculate the nucleation rate J (in number of nuclei per area per time) with the following expressions:[16]

$$J = A\exp\left(\frac{-16\pi(\sigma F_{\mathrm{eff}}/k_{\mathrm{B}}T_{\mathrm{surf}})^3(\bar{M}v_{\mathrm{l}}/N_{\mathrm{A}})^2}{3\{\ln[P_{\mathrm{v}}/P_{\mathrm{sat}}(T_{\mathrm{surf}})]\}^2}\right)$$

where

$$A = \left(\frac{2\sigma F_{\mathrm{eff}}N_{\mathrm{A}}}{\pi\bar{M}}\right)^{1/2}\left(\frac{P_{\mathrm{v}}}{RT_{\mathrm{surf}}}\right)^{5/3}\left(\frac{N_{\mathrm{A}}}{\bar{M}}\right)^{2/3}v_{\mathrm{l}}F_{\mathrm{eff}}\left(\frac{1-\cos\theta_{\mathrm{eff}}}{2}\right)$$

and

$$F_{\mathrm{eff}} = \frac{2-3\cos\theta_{\mathrm{eff}}+(\cos\theta_{\mathrm{eff}})^3}{4}$$

Here, θ_{eff} is the effective contact angle. θ_{eff} is expected to be much smaller than the intrinsic contact angle of pPFDA ($\theta_{\text{int}} = 119.2°$, see **Methods**) because the energetic barrier to nucleation within cavities is significantly lower than that on a flat surface.[18] In the expression for J, N_{A} is the Avogadro's number, $\bar{M}$ is the molecular weight, and k_{B} is the Boltzmann constant.

The volumetric nucleation rate, i.e. the total volume of nuclei per area per time, is obtained by multiplying the nucleation rate J with the volume of each nucleus. We calculate the volume of each nucleus V_{nucl} at the critical nucleation diameter d_{crit} and assuming a spherical cap shape with the surface at the intrinsic water-pPFDA contact angle $\theta_{\text{int}} = 119.2°$ using the following expression:

$$V_{\text{nucl}} = \frac{\pi}{3}\left(\frac{d_{\text{crit}}}{2}\right)^3 (2 + \cos\theta_{\text{int}})(1 - \cos\theta_{\text{int}})^2$$

We find that for an effective contact angle $\theta_{\text{eff}} = 48°$, a transition in the volumetric nucleation rate is seen around a subcooling of 1.5 K (**Supplementary Figure 27a**).

Cavity filling timescale

The structure of boehmite consists of cavities sided by nanowalls (right inset in **Figure 1a**). The time for nucleation to fill the cavity τ_{fill} can therefore be calculated as follows:

$$\tau_{\mathrm{fill}} = \frac{V_{\mathrm{cav}}}{J A_{\mathrm{cav}} V_{\mathrm{nucl}}}$$

where V_{cav} and A_{cav} are the volume and surface area of a cavity respectively. As V_{cav} and A_{cav} scale with the characteristic length scale of the cavity L_{cav} as $V_{\mathrm{cav}} \sim L_{\mathrm{cav}}^3$ (the height of boehmite nanostructures is at the same scale[19]) and $A_{\mathrm{cav}} \sim L_{\mathrm{cav}}^2$, we then calculate the cavity filling timescale τ_{fill} as a function of L_{cav} as follows:

$$\tau_{\mathrm{fill}} \sim \frac{L_{\mathrm{cav}}^3}{J L_{\mathrm{cav}}^2 V_{\mathrm{nucl}}} = \frac{L_{\mathrm{cav}}}{J V_{\mathrm{nucl}}}$$

We compute the range of L_{cav} for boehmite by measuring the projected area of each cavity in an SEM image using the polygon tool in ImageJ. L_{cav} is then taken as the square root of the measured projected area. The distribution of L_{cav} for boehmite is shown in **Figure 3c** (cavity size).

Substituting the respective equations, the timescale to fill the nanoscale cavities of boehmite is estimated to be 0.1 – 1 ms (**Supplementary Figure 27b**).

S12. Effects from droplet size mismatch

A smaller droplet has a higher Laplace pressure and a lower mass, enabling it to complete its coalescence in a shorter time compared to the larger droplet, and resulting in motion which appears to be tangential as the smaller droplet is apparently coalesced "into" the larger one. However, if the two droplets are considered as a single system, without roaming, there is little net tangential movement of its centre of mass after coalescence, as we find in **Supplementary Figure 16**. In this section, we show that tangential momentum generation is minimal from the coalescence of size-mismatched droplets.

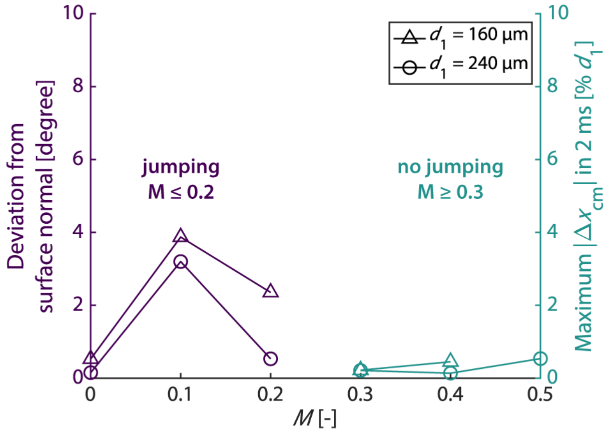

Supplementary Figure 28: Coalescence of size-mismatched droplets. Left y-axis: Jumping occurs when mismatch $M \leq 0.2$. The deviation from surface normal of the jumping motion is shown. Minimal deviation (< 4°) is observed. Right y-axis: No jumping occurs when $M \geq 0.3$. The maximum absolute centre-of-mass displacement in the in-plane x-direction in the 2 ms simulated is shown, normalised by the diameter of the smaller droplet d_1. Minimum displacement (< 1%) is observed. Source data are provided as a Source Data file.

Through numerical simulations in **Supplementary Figure 28** we predict the direction of motion of the liquid body after the coalescence of two droplets of different mismatch ratios, defined as $M = (d_2 - d_1)/(d_1 + d_2)$, where d_1 and d_2 are the diameters of the smaller and larger droplets respectively. Mismatch ratios from 0 to 0.5 are tested for d_1 = 240 µm and 0 to 0.4 are tested for d_1 = 160 µm. For both d_1, no jumping departure from the surface is observed for mismatch ratios of 0.3 or above.

For cases in which there is jumping departure ($M \leq 0.2$), we report the deviation from surface normal of the jumping motion. Our cases tested record deviations smaller than 4° (left y-axis of **Supplementary Figure 28**), in line with a previous experimental study on CuO nanostructured superhydrophobic surfaces[20] which reported a maximum deviation of 3.8°. For cases in which there is no jumping departure ($M \geq 0.3$), we report the maximum absolute centre-of-mass displacement in the in-plane x-direction in the 2 ms simulated, normalised by d_1 of the respective case. Our cases tested record maximum normalised displacements smaller than 1% (right y-axis of **Supplementary Figure 28**). It is therefore clear that substantial tangential momentum cannot be generated from size mismatch at the droplet level.

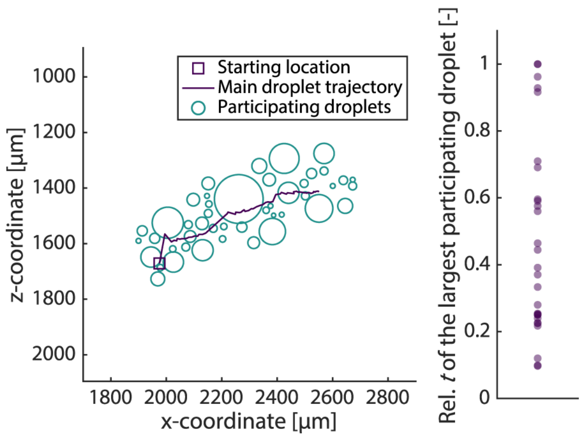

Supplementary Figure 29: Left: Participating droplets distribution of a roaming event. Right: The relative time of the largest participating droplet in each measured roaming event. Source data are provided as a Source Data file.

At the event level, there is as well no observable trend in participating droplet sizes. The largest participating droplet of a roaming event can be at any location along its trajectory. The left panel in **Supplementary Figure 29** illustrates the distribution of participating droplets of a roaming event. For this particular event, the largest participating droplet is at the middle of the trajectory. The right panel plots the distribution of the time of the largest participating droplet in each measured roaming event, relative to the duration of the event. For example, for a relative time of 1, the coalescence of the largest participating droplet occurs at the end (in terms of time) of the roaming event. The scattered distribution found in the right panel indicates that a roaming event is not driven by a trend in participating droplet sizes. Therefore, substantial tangential momentum is not generated by droplet size mismatch at the event level as well.

S13. Stages of x-momentum generation

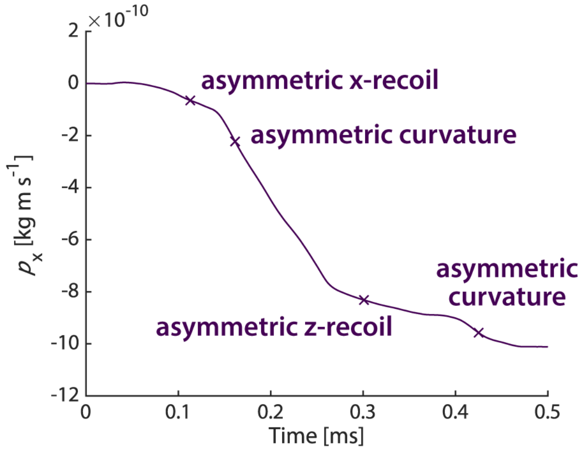

Supplementary Figure 30: x-momentum for the first 0.5 ms. Stages of momentum generation are seen. See also **Figure 4b** and **Supplementary Movie 6**. Source data are provided as a Source Data file.

We plot the variation of x-momentum for the first 0.5 ms in **Supplementary Figure 30**. Tangential momentum generation roughly follows two rates. Asymmetric recoil, originating from wettability difference, generates less momentum compared to asymmetric curvature, which originates from the elongation in the y-direction due to symmetry breaking by the substrate.

S14. Dewetting and the efficiency in kinetic energy conversion

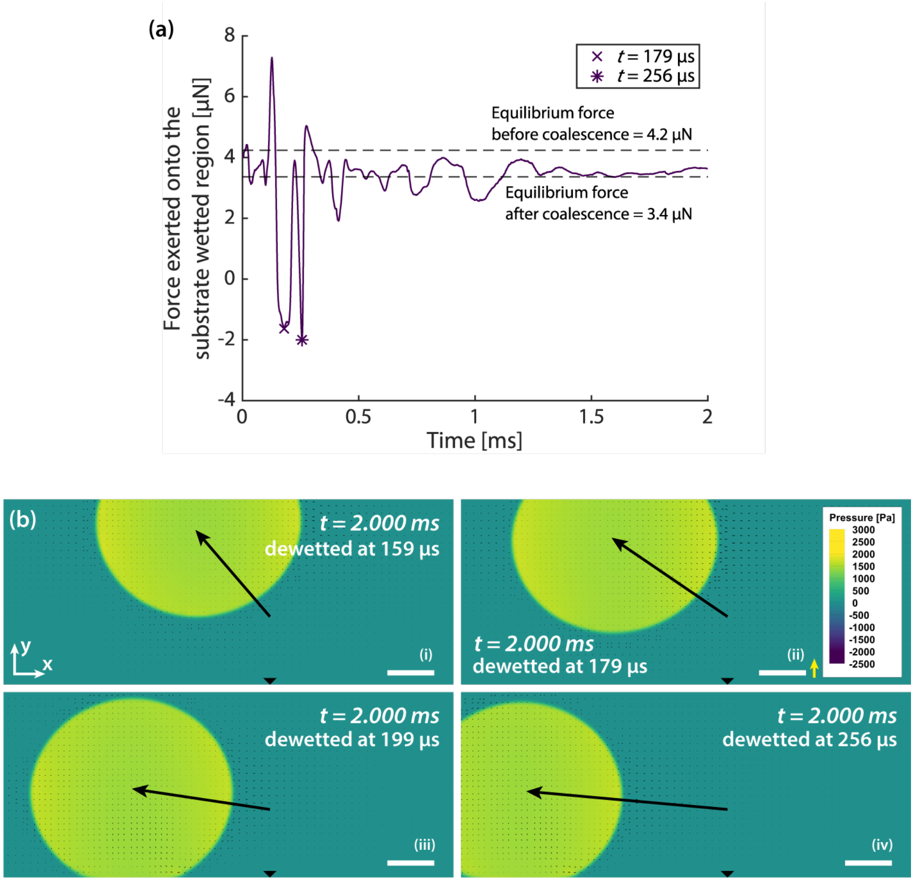

Supplementary Figure 31: (a) Force exerted onto the wetted area when there is no dewetting throughout the 2 ms (case in **Figure 4b**). Due to Laplace pressure, the equilibrium forces are non-zero, i.e. the liquid body exerts a force onto the wetted area from the curvature of the interface despite the lack of gravity. The force reported reflects a full circular wetted region, taking domain symmetry into account. (b) Droplet location at 2 ms for different dewetting times. All departures contain a substantial tangential component. Black arrows indicate the direction of motion. Markers carry the same meaning as in **Figure 4b**. Source data are provided as a Source Data file.

When two droplets coalesce and the base area of one of them is wetted, a pulling force is exerted onto the wetted region at some point during the coalescence. **Supplementary Figure 31a** displays the force on this region when there is no dewetting throughout 2 ms (case in **Figure 4b**).

In the beginning, a force of ≈ 4.2 μN is exerted. It is due to the Laplace pressure of a 160 μm diameter droplet on the wetted region:

$$\text{Force} = (2\sigma/r_{\text{curv}})A_{\text{wetted}}$$

where $\sigma = 0.072\ \text{N m}^{-1}, r_{\text{curv}} = 160/2 = 80\ \mu\text{m}$ is the radius of curvature and $A_{\text{wetted}} = \pi\left(\frac{160}{2}\sin 160°\right)^2$ is the area of the wetted region.

Then, as the two droplets coalesce, a pulling force is exerted onto the wetted region. There are two peaks, as indicated by a cross and an asterisk in **Supplementary Figure 31a**. In **Figure 5**, we choose to dewet at 179 μs and substantial x-displacement in the departure is seen.

As there is no dewetting for the case corresponding to the plot in **Supplementary Figure 31a**, the two droplets complete coalescence and come to rest on top of the wetted region. At this point it exerts a lower force than in the beginning, as the new radius of curvature ($r_{\text{curv}} = 101\ \mu\text{m}$) is larger with the combined volume of the two droplets. Using the equation above, it can be calculated that the equilibrium force after coalescence with the new radius of curvature is 3.4 μN. In **Supplementary Figure 31a** we can observe that the force from our simulation converges to this value.

Supplementary Figure 31b displays the location of the droplet at 2 ms for different dewetting times. We choose to dewet at the two peaks in **Supplementary Figure 31a**, as well as two additional times at 159 and 199 μs. It can be seen that the later is the dewetting, the higher the x-displacement is. **Supplementary Figure 32** displays the kinetic energy of the translational motion of the centre of mass compared to the total kinetic energy, for different dewetting times and when there is no dewetting. Similarly, the later is the dewetting, the closer KE_{cm} is to KE_{tot}, indicating a higher energy conversion efficiency. This arises from the fact that for wetting cases,

the y-centre of mass is consistently lower, and the liquid body is closer to the surface. As it oscillates, there is stronger symmetry breaking than the reference case where the droplet is departing normally from the surface earlier in the coalescence process. The exact dewetting time is dependent on the adhesion strength, but a general feature is that significant tangential momentum is generated to result in a much higher angle of motion than the 4° deviation from surface normal in conventional droplet jumping without adhesion asymmetry (**Supplementary Information S12**). In **Figure 5a**, it is 56.6°. In rare cases, if the main droplet is not intercepted by droplets on the surface after dewetting, in-plane roaming is prevented and it could depart the surface with motion at these large angles. This can be seen experimentally, for example, in Clips 2 and 4 of **Supplementary Movie 2**: At a subcooling higher than the transition subcooling, the droplet jumps away from the surface at large angles with significant tangential momentum already at the moment of departure, without first hovering in the vapour over their original locations and then gaining tangential momentum through the leftward vapour flow and downward gravity.

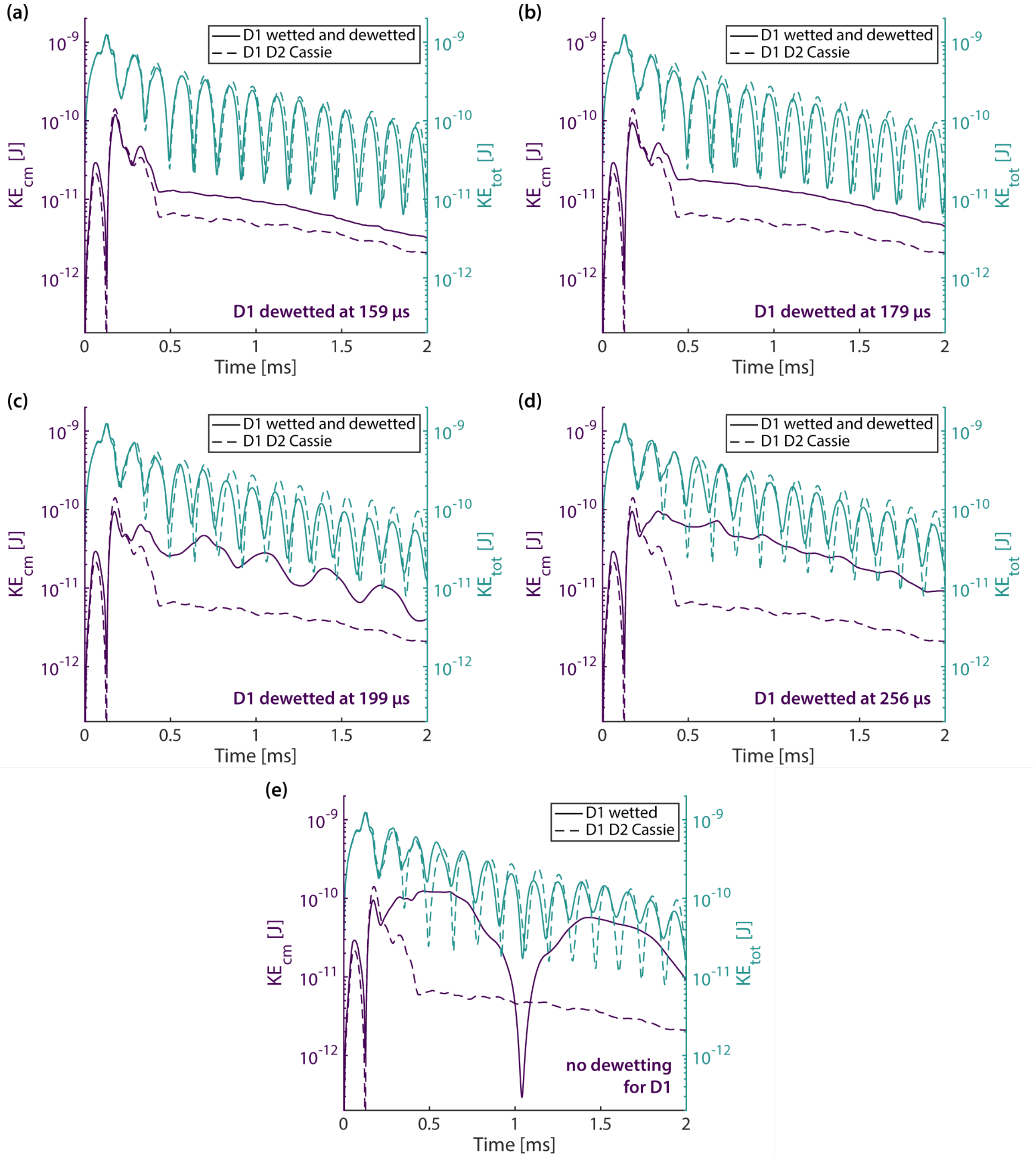

Supplementary Figure 32: Kinetic energy of the translational motion of the centre of mass KE_{cm} and the total kinetic energy KE_{tot} for (a) to (d) different dewetting times, and (e) no dewetting. The kinetic energy reported reflects full spherical droplets, taking domain symmetry into account. Source data are provided as a Source Data file.

S15. Setup of numerical simulation cases

Numerical studies are performed with Ansys Fluent 2021 R2 on the Euler computing cluster of ETH Zurich. This section outlines the setup of simulation cases involving two droplets, with or without droplet size mismatch.

Domain and meshing

The three-dimensional computational domain is created and meshed with hexahedra in Ansys ICEM CFD 2021 R2. An example can be seen in **Figure 4a**.

The domain is in the shape of a cuboid and split along a symmetry plane, where the centre-to-centre line of the two droplets lie. Nanostructures are not explicitly included in the geometry, but modelled as a flat superhydrophobic surface on the x-z plane. The symmetry plane is normal to the z-axis.

The domain size is specified as multiples of the mean droplet radius. The x-, y- and z-dimensions are (12,10,6) $\bar{r}$ respectively,[21] where $\bar{r}$ is the mean radius of the two droplets. Cells are cubes with equal edge lengths Δh in all dimensions. The maximum global cell size is specified as 10 cells per radius of the smaller droplet. For cells around the liquid-vapour interface and within the droplet(s), their size is refined to 40 cells per radius of the smaller droplet,[21] through solution-based adaptation described later.

Model and fluid properties

The multiphase flow is modelled with the volume of fluid (VOF) method. Two discrete phases are specified, namely water vapour and liquid water. The density and viscosity for water vapour are 0.021904 kg m^{-3} and 9.6718×10^{-6} kg m^{-1} s^{-1}, and the density and viscosity for liquid water are 997.24 kg m^{-3} and 9.0904×10^{-4} kg m^{-1} s^{-1}, corresponding to their saturation properties at 30 mbar, the pressure maintained in the experiments. These properties are obtained with CoolProp.[22] The surface tension is taken to be 0.072114 N m^{-1}, provided by The International Association for the Properties of Water and Steam,[23] and similarly at saturation at 30 mbar. The continuum surface force model[24] is used to model surface tension and gravity is disabled.

Boundary conditions

The superhydrophobic surface is modelled as a no-slip wall with a specified contact angle. Symmetry is specified at the symmetry plane of the droplets. The three vertical boundaries are specified as pressure inlets with a gauge pressure of zero and normal flow direction. Similarly, the top horizontal boundary is specified as a pressure outlet with a gauge pressure of zero. Gauge pressures are computed using an operating pressure of 30 mbar.

Solver

A double-precision pressure-based transient solver is used. The PISO scheme is specified for pressure-velocity coupling. For spatial discretisation, gradients are computed using the least squares cell based method. The scheme for pressure is PRESTO!, second order upwind for momentum, and geo-reconstruct for volume fraction. For temporal discretisation, the first order implicit scheme is used. To determine convergence, the residuals of continuity and all three velocities should reach 10^{-6} to proceed to the next time step. The total mass balance across all flow boundaries is monitored as well.

Mesh adaptation

A mesh adaptation strategy is adopted to refine the mesh around and within the droplet(s) to resolve and capture dynamics at small scales for the droplet(s). A cell marked for refinement satisfies at least one of the two criteria:

1) The gradient of the liquid water volume fraction is larger than 6% of the global maximum, so that the cell is refined if it is close to any liquid/vapour interface; or
2) The liquid water volume fraction is larger than the cut-off value of 10^{-6}, so that the cell is refined if it is occupied with liquid water.

Cells marked for refinement are refined to 1/4 of their original edge length, corresponding to 1/64 of their original cell volume. As the solution proceeds and a cell becomes occupied with only water vapour and far away from any liquid/vapour interface, it is coarsened back to its original edge length. A cell marked for coarsening has to satisfy both criteria:

1) The gradient of the liquid water volume fraction is smaller than 5% of the global maximum, so that the cell is away from any liquid/vapour interface; and
2) The liquid water volume fraction is smaller than the cut-off value of 10^{-6}, so that the cell is occupied with water vapour only.

The mesh is pre-adapted during solution initialisation using the criteria above. During calculation, the adaptation is updated every two time steps based on the instantaneous solution flow field, refining and coarsening the concerned cells.

Solution initialisation

The entire flow field is first initialised with zero gauge pressure, velocity and liquid water volume fraction. Then, cells which constitute a droplet are patched with a liquid water volume fraction of one. The mesh is adapted repeatedly with the criteria above until no more cells are marked for refinement or coarsening. However, note that this resulting mesh is adapted from a liquid droplet patched onto the initial coarse mesh, i.e. the droplet is approximated by a coarse interface to start with. As mesh adaptation does not alter the flow field, the droplet remains coarse albeit on the refined, adapted mesh. If time marching begins here, it would take a few time steps for the coarse droplet and its liquid-vapour interface to relax into the refined mesh, distorting the solution. Therefore, to ensure that the droplet is patched onto a sufficiently refined mesh so that its contour is well resolved in the beginning, we repeat the initialisation, patching and adaptation procedure until the mesh adaptation criteria no longer mark any cells for refinement or coarsening after patching the liquid droplet cells.

Solution computation and time advancement

With the domain and solution properly initialised, the case can proceed to computation. To capture all dynamics, we select a time step size smaller than the timescale of relevant physical phenomena, derived at the length scale of the refined cell size. Among surface tension, convective flow, and viscous dissipation, the timescale for surface tension is found to be the smallest, computed as follows:[24]

$$t_\sigma = \sqrt{\frac{\bar{\rho}(\Delta h)^3}{2\pi\sigma}}$$

where $\bar{\rho}$ is the mean density of the two phases, Δh is the cell edge length, and σ is the surface tension. For a case in which the smaller droplet has a radius of 80 μm, the adapted cell edge length is 2 μm and the resulting surface tension timescale is 94 ns. A time step size of 90 ns is then specified for the computation.

Result reporting and postprocessing

During computation, results including droplet centre of mass, momentum and kinetic energy are reported and saved every time step. Selected variables of the flow field solution is saved at least every 1 μs and the entire flow field solution is saved at least every 10 μs. Postprocessing is performed in Tecplot 360 EX 2021 R2 and MATLAB R2022b (MathWorks).

Computation of variables of interest

Several variables are computed during the simulation and in postprocessing. We report the expression for the computation of a number of them in the following.

1) Centre-of-mass location

The x-coordinate of the centre of mass of the liquid body is calculated as follows:

$$x_{\mathrm{cm}} = \frac{\int_{\Omega} \rho \alpha x \, d\Omega}{\int_{\Omega} \rho \alpha \, d\Omega}$$

where $d\Omega$ is the volume of a cell in the domain, ρ is the density of the liquid, x is the x-coordinate of the cell and α is the volume fraction of the liquid of the cell. The integrals integrate over the entire computational domain Ω. The y- and z-coordinates of the centre of mass of the liquid body are calculated similarly, replacing x with y and z respectively.

2) Momentum

The total x-momentum of the liquid body is calculated as follows:

$$p_{\mathrm{x}} = \int_{\Omega} v_{\mathrm{x}} \rho \alpha \, d\Omega$$

Where v_{x} is the velocity in the x-direction of the cell. The total y- and z-momentum of the liquid body are calculated similarly, replacing v_{x} with v_{y} and v_{z} respectively.

3) Total kinetic energy

The total x-kinetic energy of the liquid body is calculated as follows:

$$\mathrm{KE}_{\mathrm{x,\,tot}} = \int_{\Omega} \frac{1}{2} \rho \alpha v_{\mathrm{x}}^{2} \, d\Omega$$

The total y- and z-kinetic energies of the liquid body are calculated similarly, replacing v_{x} with v_{y} and v_{z} respectively. The total kinetic energy of the liquid body is the sum of all three directions.

4) Kinetic energy of the translational motion of the centre of mass

The x-kinetic energy of the translational motion of the centre of mass of the liquid body is calculated as follows:

$$\mathrm{KE}_{\mathrm{x,\,cm}} = \frac{1}{2} \left(\int_{\Omega} \rho \alpha \, d\Omega \right) \left(\frac{dx_{\mathrm{cm}}}{dt} \right)^{2}$$

The integral is the total mass of the liquid body and dx_{cm}/dt is the velocity of the centre of mass of the liquid body in the x-direction. The y- and z-kinetic energies of the translational motion of the centre of mass of the liquid body are calculated similarly, replacing x_{cm} with y_{cm} and z_{cm} respectively. The kinetic energy of the translational motion of the centre of mass is the sum of all three directions.

S16. Roaming on hierarchical surfaces

Roaming events shown in this work are on solely nanostructured surfaces, namely superhydrophobic boehmite nanowalls, copper(II) hydroxide nanoneedles, and titanium dioxide nanorods. This has eliminated effects from microstructures. In particular, any apparent tangential droplet motion from the jumping of droplets on the side walls of microstructure cavities is not possible. All tangential motion has to therefore result from roaming effects and asymmetry in droplet adhesion, as we have shown and explained.

Nevertheless, roaming on hierarchical surfaces might be possible. In **Supplementary Figure 33**, a hierarchical aluminium surface is shown. Microstructures are imparted onto the substrate by etching with iron(III) chloride, before nanostructuring. A droplet travelling in-plane can be seen in **Supplementary Figure 34**.

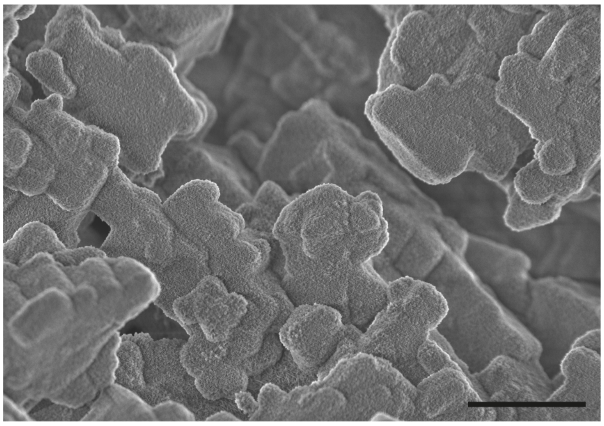

Supplementary Figure 33: Hierarchically structured aluminium. The microstructures are overlayed with nanostructures. Scale bar: 5 μm.

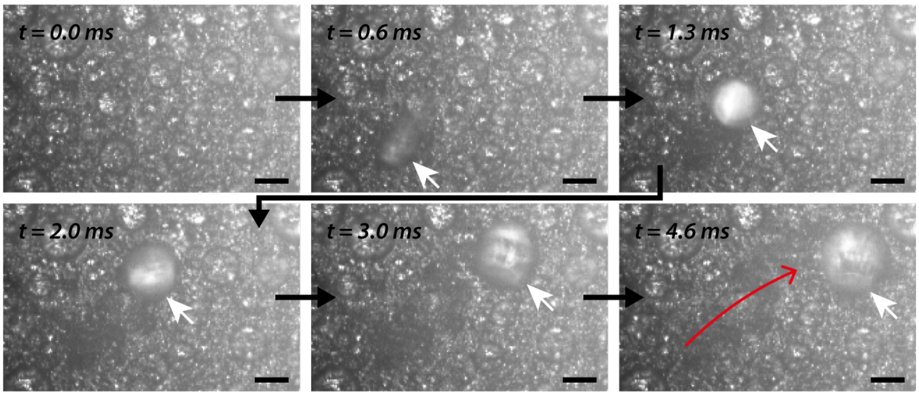

Supplementary Figure 34: Droplet travelling in-plane on the hierarchically structured surface. White arrow points to the main droplet. Red arrow indicates approximate trajectory. Scale bar: 100 μm.

The event in **Supplementary Figure 34** resembles very closely the roaming behaviour we observe on solely nanostructured surfaces. However, the presence of microstructures interferes with the interpretation because it is no longer possible to decouple the effects from (1) asymmetric adhesion and (2) jumping from side walls. The tangential momentum observed may be a result of both. Therefore, in our work, we have focused exclusively on solely nanostructured surfaces.

We speculate that roaming on hierarchical surfaces may provide further improvement in heat transfer efficiency, due to (1) the larger surface area for hierarchically structured surfaces than solely nanostructured surfaces, and (2) a potentially higher surface area renewal rate due to the additional tangential momentum generation mechanism. More nuclei can form in a given period of time.

References

1. Donati, M. *et al.* Sprayable Thin and Robust Carbon Nanofiber Composite Coating for Extreme Jumping Dropwise Condensation Performance. *Adv. Mater. Interfaces* **8**, 2001176 (2021).
2. Tripathy, A. *et al.* Ultrathin Lubricant-Infused Vertical Graphene Nanoscaffolds for High-Performance Dropwise Condensation. *ACS Nano* **15**, 14305–14315 (2021).
3. Sharma, C. S., Lam, C. W. E., Milionis, A., Eghlidi, H. & Poulikakos, D. Self-Sustained Cascading Coalescence in Surface Condensation. *ACS Appl. Mater. Interfaces* **11**, 27435–27442 (2019).
4. Taylor, J. R. *An Introduction to Error Analysis: The Study of Uncertainties in Physical Measurements*. (University Science Books, Sausalito (Calif.), 1997).
5. Holman, J. P. *Heat Transfer*. (McGraw-Hill, New York, 2002).
6. Qu, X. *et al.* Self-propelled sweeping removal of dropwise condensate. *Appl. Phys. Lett.* **106**, 221601 (2015).
7. Yan, X. *et al.* Hierarchical Condensation. *ACS Nano* **13**, 8169–8184 (2019).
8. Boreyko, J. B. & Chen, C.-H. Self-Propelled Dropwise Condensate on Superhydrophobic Surfaces. *Phys. Rev. Lett.* **103**, 184501 (2009).
9. Muir, D. DylanMuir/ReadImageJROI. https://github.com/DylanMuir/ReadImageJROI (2023).
10. Dorrer, C. & Rühe, J. Wetting of Silicon Nanograss: From Superhydrophilic to Superhydrophobic Surfaces. *Adv. Mater.* **20**, 159–163 (2008).
11. Wen, R. *et al.* Hierarchical Superhydrophobic Surfaces with Micropatterned Nanowire Arrays for High-Efficiency Jumping Droplet Condensation. *ACS Appl. Mater. Interfaces* **9**, 44911–44921 (2017).

12. Song, J. *et al.* Inhibition of condensation-induced droplet wetting by nano-hierarchical surfaces. *Chem. Eng. J.* **460**, 141761 (2023).

13. Stamatopoulos, C. *et al.* Droplet Self-Propulsion on Superhydrophobic Microtracks. *ACS Nano* **14**, 12895–12904 (2020).

14. Wang, K. *et al.* Numerical Simulation of Coalescence-Induced Jumping of Multidroplets on Superhydrophobic Surfaces: Initial Droplet Arrangement Effect. *Langmuir* **33**, 6258–6268 (2017).

15. Li, S. *et al.* Durable, Ultrathin, and Antifouling Polymer Brush Coating for Efficient Condensation Heat Transfer. *ACS Appl. Mater. Interfaces* **16**, 1941–1949 (2024).

16. Carey, V. P. *Liquid-Vapor Phase-Change Phenomena: An Introduction to the Thermophysics of Vaporization and Condensation Processes in Heat Transfer Equipment*. (CRC Press, Taylor & Francis Group, Boca Raton, 2020).

17. Mouterde, T. *et al.* Antifogging abilities of model nanotextures. *Nat. Mater.* **16**, 658–663 (2017).

18. Aili, A., Ge, Q. & Zhang, T. How Nanostructures Affect Water Droplet Nucleation on Superhydrophobic Surfaces. *J. Heat Transf.* **139**, 112401 (2017).

19. Sharma, C. S., Combe, J., Giger, M., Emmerich, T. & Poulikakos, D. Growth Rates and Spontaneous Navigation of Condensate Droplets Through Randomly Structured Textures. *ACS Nano* **11**, 1673–1682 (2017).

20. Yan, X. *et al.* Droplet Jumping: Effects of Droplet Size, Surface Structure, Pinning, and Liquid Properties. *ACS Nano* **13**, 1309–1323 (2019).

21. Vahabi, H., Wang, W., Davies, S., Mabry, J. M. & Kota, A. K. Coalescence-Induced Self-Propulsion of Droplets on Superomniphobic Surfaces. *ACS Appl. Mater. Interfaces* **9**, 29328–29336 (2017).

22. Bell, I. H., Wronski, J., Quoilin, S. & Lemort, V. Pure and Pseudo-pure Fluid Thermophysical Property Evaluation and the Open-Source Thermophysical Property Library CoolProp. *Ind. Eng. Chem. Res.* **53**, 2498–2508 (2014).

23. Petrova, Tamara. *Revised Release on Surface Tension of Ordinary Water Substance*. http://www.iapws.org/relguide/Surf-H2O-2014.pdf (2014).

24. Brackbill, J. U., Kothe, D. B. & Zemach, C. A continuum method for modeling surface tension. *J. Comput. Phys.* **100**, 335–354 (1992).
