# Supplementary material for: Condensate droplet roaming on nanostructured superhydrophobic surfaces
Source: Nat Commun. 2025 Jan 30;16:1167. doi: 10.1038/s41467-025-56562-x (PMC11782698; doi:10.1038/s41467-025-56562-x)
Supplement: Supplementary file 2 — Description of Additional Supplementary Files [file 41467_2025_56562_MOESM2_ESM.docx]

**Description of additional supplementary files**

File Name: Supplementary Movie 1

Description: Typical roaming event

File Name: Supplementary Movie 2

Description: Local clustered multi-droplet coalescence

File Name: Supplementary Movie 3

Description: Roaming events altering direction

File Name: Supplementary Movie 4

Description: Roaming events terminating in jumping

File Name: Supplementary Movie 5

Description: Condensation modes and heat transfer coefficients at different subcooling

File Name: Supplementary Movie 6

Description: Source of tangential momentum

File Name: Supplementary Movie 7

Description: Motion for dewetting at different times

File Name: Supplementary Movie 8

Description: Dewetting during roaming
